# Potential-Switchable Viscoelasticity of Protein Nanolayers at a Liquid/Liquid Interface

**DOI:** 10.1021/acs.langmuir.5c01819

**Published:** 2025-07-01

**Authors:** Kosuke Ishii, Takeshi Ueki, Jun Nakanishi, Kazuhiro Akutsu-Suyama, Norifumi L. Yamada, Yuko Yokoyama, Tetsuo Sakka, Naoya Nishi

**Affiliations:** † Department of Energy and Hydrocarbon Chemistry, 12918 Kyoto University, Kyoto 615-8510, Japan; ‡ Research Center for Macromolecules & Biomaterials, National Institute for Materials Science (NIMS), 1-1 Namiki, Tsukuba, Ibaraki 305-0044, Japan; § Graduate School of Life Science, Hokkaido University, Kita 10, Nishi 8, Kita-ku, Sapporo 060-0810, Japan; ∥ Graduate School of Advanced Science and Engineering, Waseda University, 3-4-1 Okubo, Shinjuku-ku, Tokyo 169-8555, Japan; ⊥ Graduate School of Advanced Engineering, Tokyo University of Science, 6-3-1 Niijuku, Katsushika-ku, Tokyo 125-8585, Japan; # Neutron Science and Technology Center, Comprehensive Research Organization for Science and Society (CROSS), Tokai, Naka 319-1106, Ibaraki, Japan; ∇ Neutron Science Laboratory, Center for Integrative Quantum Beam Science, High Energy Accelerator Research Organization, Tokai, Naka 319-1106, Japan

## Abstract

Protein nanolayers (PNLs) formed at an electrochemical liquid|liquid interface between water (W) and a fluorous solvent (F) were examined by using interfacial rheological measurement (IRM) and neutron reflectometry (NR) under the externally controlled condition of the phase boundary potential differences *E*_F_^W^(= φ^W^ − φ^*F*^ + const.), where F contained a hydrophobic ionic liquid (IL) as a supporting electrolyte and W, whose pH was 7.4, contained a protein, bovine serum albumin (BSA). The IRM and NR results illuminated that both static and dynamic properties of the PNL at the electrochemical F|W interface were varied by applying *E*_F_^W^. NR found minimal *E*_F_^W^ dependence on the adsorption amount of BSA in the PNL. In contrast, IRM revealed that although the interfacial shear loss moduli *G*″ of the PNL was constant regardless of *E*_F_^W^, the interfacial shear storage *G*′ of the PNL increased dramatically at more negative *E*_F_^W^, showing a more elastic response. This difference between static and dynamic properties results from the increase in intermolecular and intramolecular interactions between BSA molecules in the PNL at more negative *E*_F_^W^ due to the accelerated denaturation of negatively charged BSA that formed complexes with IL cations accumulated on the F side of the F|W interface. The *G*′ and *G*″ reversibly responded to switching between different potentials (a positive and a negative *E*_F_^W^). These IRM results unveiled that the viscoelasticity of the PNL at the electrochemical F|W interface is reversibly potential-switchable. The present interface-specific method using the potential control is a new promising method to diversify and switch the PNL structure reversibly. The reversible structural control of the PNL would enable us to perform real-time observation of cells reacting to environmental changes at liquid|liquid interfaces.

## Graphical abstract

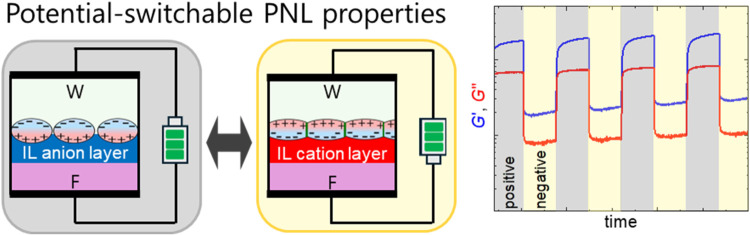

## 1 Introduction

Mechanobiology is a research field that focuses on how cells sense and respond to mechanical cues such as substrate viscoelasticity. In this context, cell culture platforms with well-defined and tunable mechanical properties have become indispensable tools for studying cellular phenotypes such as adhesion, migration, and differentiation. Against this background, hydrophobic liquid interfaces have recently emerged as a novel platform for mechanobiology. The liquid phase used as a cell scaffold must form a clear biphasic system with water, be noncytotoxic, and have a density higher than water. Since Rosenberg reported in 1964,^1^ that cells adhere to and spread on fluorinated liquids such as FC-70, numerous studies have explored cell dynamics at the interfaces of various molecular liquids, including silicone oils^2,3^ and fluorinated liquids.^4–8^ In recent years, notable biological phenomena have been reported at liquid interfaces, including selective neuronal differentiation^9^ of human mesenchymal stem cells (hMSCs) and the maintenance of their undifferentiated state.^10^ At these liquid interfaces, proteins from the culture medium spontaneously accumulate to form a protein layer with a thickness of several nm,^11,12^ so-called protein nanolayer (PNL), which acts as a mechanically robust, solid-like scaffold for cell adhesion and spreading. However, PNLs formed via spontaneous protein self-assembly are often mechanically fragile, sometimes failing to adequately support cell adhesion. To address this problem, Keese and Geaver reported a method to enhance the mechanical robustness of PNLs by anchoring them to fluorinated liquid interfaces using reactive surfactants.^13–15^ Other strategies have also been proposed, including pretreatment with certain proteins that support cell adhesion^16^ and actively denaturing proteins at the interface^17^ to reinforce the mechanical properties of PNLs.

Recently, we introduced hydrophobic ionic liquid (IL) interfaces as a new class of liquid scaffolds for cell culture.^18^ We demonstrated that certain alkylphosphonium-based ILs (and some alkylammonium-based ILs^19^) exhibit low cytotoxicity and can support cell culture at their interfaces. Similar to conventional liquid scaffolds, PNLs also form at IL interfaces, and their mechanical robustness plays a key role in cell adhesion. The apparent Young’s modulus of PNLs in the vertical direction at IL interfaces is lower than that at fluorinated liquid interfaces. Nevertheless, cell adhesion occurred at the IL interface, and it was found that the degree of cell spreading at the interface varied depending on subtle differences in the IL chemical structure.^18^ Furthermore, by leveraging the high miscibility of ILs with various (macro) molecules, we successfully modified IL-based PNLs by incorporating a cross-linked polymer to enhance the bulk mechanical properties, thereby modulating cell spreading and morphology.

In the present study, to diversify cell culture on liquid|liquid interfaces, the PNL structure at the electrochemical fluorinated liquid|water (F|W) interface was controlled by modulating the interfacial ionic composition through phase boundary potential difference switching. Given the high solubility of ILs with a fluorinated anion in fluorinated liquids,^20^ we employed an IL as a supporting electrolyte in the subphase. Interfacial rheological measurements (IRM), which have been extensively used to investigate the rheological properties of PNLs at nonelectrochemical liquid|liquid interfaces,^8,21–27^ were applied here to the electrochemical liquid|liquid interface. Neutron reflectometry (NR) was utilized to characterize the PNL at the electrochemical F|W interface. While NR has been previously used to investigate PNLs at electrode|W interfaces^28,29^ and those at nonelectrochemical oil (O)|W interfaces,^8,26,30,31^ as well as electric double layers at electrochemical O|W^32^ and F|W^33^ interfaces, to the best of our knowledge, NR has never been applied to examine the potential-dependent structure of PNLs at electrochemical liquid|liquid interfaces. In this study, we demonstrate that IRM and NR are powerful tools for probing the static and dynamic properties of PNLs under electrochemical conditions. Unlike chemical modifications used in previous studies^3,13,14,34^ to enhance the mechanical properties of PNLs, our approach enables interfacial-specific reinforcement of PNLs via physical (electrochemical) methods. Because IL interfaces have higher polarity compared to conventional subphases such as silicone oils and some fluorinated liquids, PNLs formed via interfacial tension-driven assembly tend to be mechanically weaker. However, we show that electrochemical modulation can improve the mechanical robustness of PNLs, potentially overcoming this limitation. Furthermore, by utilizing a highly switchable electrochemical stimulus, we achieve a reversible modulation of interfacial elasticity across an order of magnitude, with high temporal resolution. The creation of cell scaffolds capable of delivering reversible mechanical stimuli and enabling real-time observation of cell dynamics at interfaces represents a growing trend in mechanobiology.^35–38^ Our system offers a qualitatively new approach to liquid-based cell scaffolds, providing a means to apply localized mechanical stimuli to cells at liquid interfaces in a manner distinct from conventional methods.

## 2 Experimental Section

### 2.1 Materials

The fluorinated liquid (F) used was 1,1,1,2,2,3,4,5,5,5-decafluoro-3-methoxy-4-(trifluoromethyl)­pentane (DMTMP, TCI, Figure 1). As the supporting electrolyte in F, trihexyltetradecylphosphonium bis­(nonafluorobutanesulfonyl)­amide ([THTDP^+^]­[C_4_C_4_N^−^]), which is a hydrophobic IL, was dissolved at 2.5 mM, as was in our previous study on the electric double layer at the F|W interface.^33^ [THTDP^+^]­[C_4_C_4_N^−^] (Figure 1) was prepared from [THTDP^+^]­Cl^−^ (Sigma-Aldrich) and Li^+^[C_4_C_4_N^−^] (Mitsubishi Materials Electronic Chemicals) and purified by using the same methods as those described elsewhere.^39^

**Figure 1:**
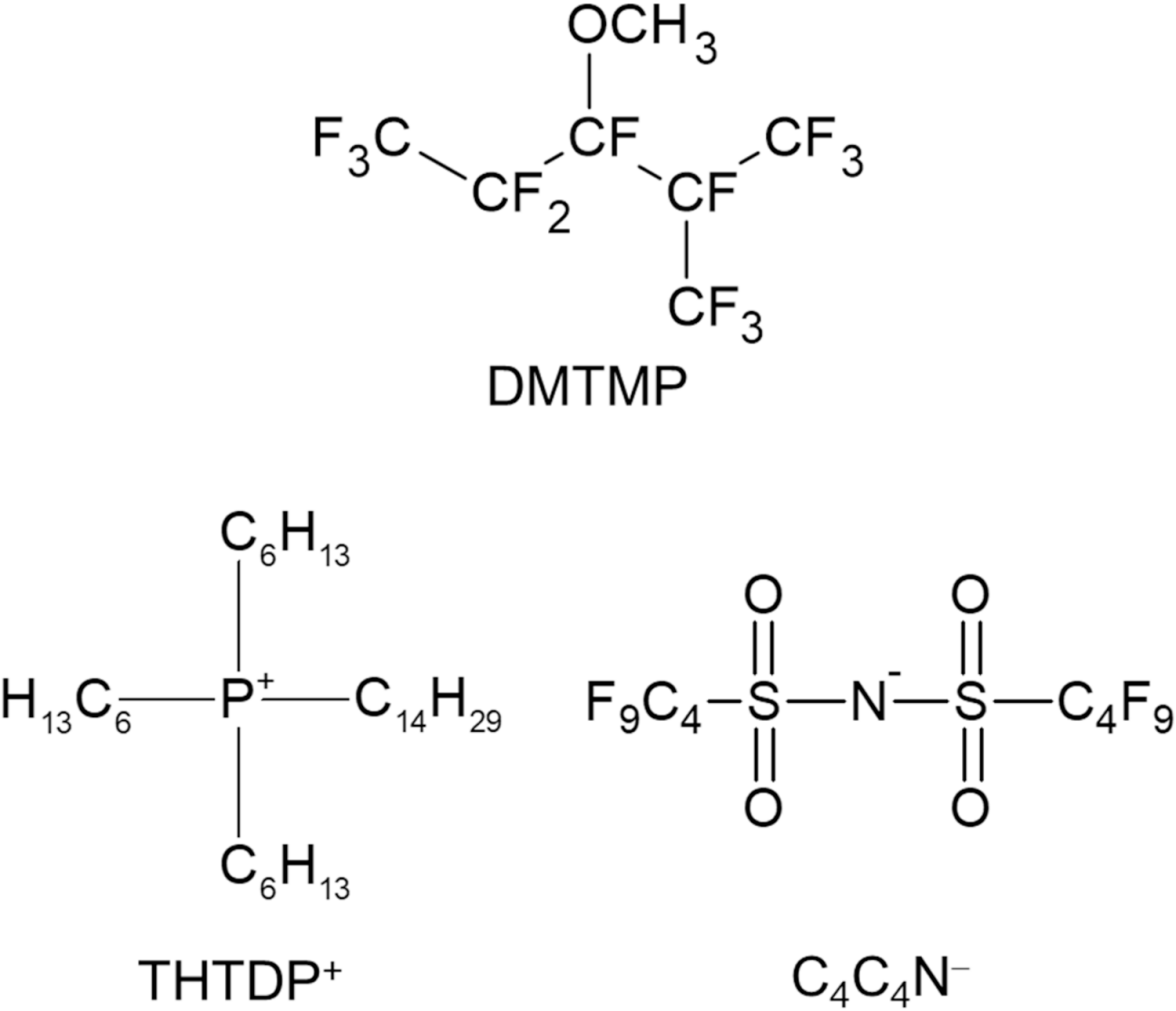
Structures of DMTMP and [THTDP^+^]­[C_4_C_4_N^−^].

For IRM, a phosphate buffer (PB, pH 7.4) was prepared by dissolving 0.2 mM Na_2_HPO_4_·2H_2_O and 0.2 mM NaH_2_PO_4_·12H_2_O (Wako) in H_2_O (Milli-Q). With this PB, a bovine serum albumin (BSA) solution for IRM was prepared which contained 1 mg/mL BSA (Wako, first grade, pH 5.2) and 1 mM NaCl (Kishida). The final concentration of BSA in W_7.4_ (the pH 7.4 buffered W phase) for IRM was 0.5 mg/mL, half of that in the above BSA solution because the two-phase system was constructed with a 1 mM NaCl solution without BSA first and then an equal amount of the BSA solution was added to start the BSA adsorption (see Section 2.3 for the detail).

For NR, a PB (pH 7.4) was prepared by dissolving 0.2 mM Na_2_HPO_4_ and 0.2 mM NaH_2_PO_4_ (Wako) in D_2_O (Silanes, >99.9%). A tartaric acid buffer (pH 2.6) was also prepared by dissolving 0.8 mM C_4_H_4_Na_2_O_6_ (Wako) and 1 mM NaCl in D_2_O. With the PB and the tartaric acid buffer, two BSA solutions with pH 7.4 and 2.6, were prepared, both containing 1 mg/mL BSA (Wako, Crystallized) and 1 mM NaCl (Kishida). The final concentration of BSA both in W_7.4_ and W_2.6_ was 0.5 mg/mL, because of the dilution similar to the IRM case (see above and Section 2.2). It is noted that the isoelectric point of BSA in a 1 mM NaCl solution is pH 4.8,^40^ indicating that BSA is negatively and positively charged at pH > 4.8 and pH < 4.8, respectively.

### 2.2 Neutron Reflectometry

NR was performed using a horizontal-type neutron reflectometer, SOFIA, at BL16 of the Materials and Life Science Experimental Facility (MLF) of the Japan Proton Accelerator Research Complex (J-PARC).^41,42^ The *q* range was 0.02−0.08 Å^−1^ (the incident angles were 0.4/1.0°). The NR cell used was the same as our previous NR study for the electrochemical F|W interface.^33^ The F|W interface was formed in the following way. First, 15 mL of 1 mM NaCl D_2_O solution (upper phase) was gently placed in a quartz cell, and then 8 mL of F (lower phase) was slowly injected into the cell from the bottom using a syringe pump to form the F|W interface. Then, 15 mL of the BSA solution (pH 7.4 or 2.6) was added to W to start the BSA adsorption at the F|W interface. NR was performed 1 h after the addition of the BSA solution when the BSA adsorption was saturated, judging from interfacial tension measurements (Figure S1-2). The bulk concentration of BSA in the W was 0.5 mg/mL, which is among the concentration range used in previous studies on the PNL formation of BSA at liquid/liquid interfaces.^12,43^

The reflectivity data were analyzed using a one-slab model taking into account the interface layer (L) on the W side of the F|W interface, which corresponds to the PNL on the interface and does not take into account the molecules/ions in F: THTDP^+^, C_4_C_4_N^−^, and DMTMP. The scattering length density (SLD) changes on the F side of the interface are regarded as negligible, from the fact that the number density of THTDP^+^, C_4_C_4_N^−^, and DMTMP at the interface was not so high (<3 × 10^−3^ Å^−2^ from our previous NR study on the electric double layer at the F|W interface)^33^ compared with that of BSA (2 × 10^−4^ Å^−2^ roughly estimated from a reported adsorption amount of BSA at the O|W interface, 2 mg/m^2^)^30^ while their scattering lengths (THTDP^+^: −36.5, C_4_C_4_N^−^: 193.2, and DMTMP: 114.6 fm)^33^ are 2 orders of magnitude smaller than that of BSA (*b*_BSA_ = 26000 fm, calculated from the structure of BSA taken from NCBI data sets^44^ by using a calculator provided by MULCh^45^). This indicates that THTDP^+^, C_4_C_4_N^−^, and DMTMP do not substantially affect the SLD in the PNL. The following conditions were used for fitting. The SLD of F and W (ρ_F_ = 3.13, ρ_W_ = 6.16 × 10^−6^ Å^−2^) were set to the values obtained from the NR at the F|air and W|air interfaces (see S2 in Supporting Information). The surface roughness between L-F (namely, the F|W interface), σ_L-F_ was fixed to the values of σ_A_ estimated from the capillary wave theory^46,47^ (see S3). To determine the surface roughness between W-L σ_W-L_, we evaluated two models: one with σ_W-L_ fixed at the same value as σ_L-F_ and the other with nonfixed σ_W-L_. The corrected Akaike Information Criterion (AICc),^48^ a measure of the model likelihood, showed that the fixed σ_L-F_ model was more likely (Table S4−1). In the following, we discuss the NR results obtained employing a one-slab model with the fixed σ_W-L_ even though both fitted results were similar. The fitted results without fixed σ_W-L_ are shown in Figure S4−2 and Table S4−2.

The value of *A*_BSA_ (= *d*(*b*_BSA_ × *n*_BSA_−ρ_F_)), with the number density of BSA in L *n*_BSA_ and thickness of L *d*, reflects the adsorption amount of BSA and was extracted by using a code made by ourselves used in our previous papers.^33,49,50^ In the following section, *A*_BSA_ was used as a parameter of the accumulated BSA to the interface.

### 2.3 Interfacial Rheological Measurements

The viscoelasticity of the PNL at the electrochemical F|W interface was measured by using a rheometer (HR20, TA Instruments) equipped with a Pt-Ir ring wire in a double wall-ring geometry (Figure 2). The cross-section of the ring wire was square with a diagonal of 1 mm. A Pt coil and Ag/AgCl wire were placed in F as the counter and quasi-reference electrode, respectively, which were covered with PTFE tubes not to contact with W. An Ag/AgCl coil was placed in W as the counter/reference electrode. The potential difference between the Ag/AgCl wire in F and the Ag/AgCl coil in W *E*_F_^W^(= φ^W^ − φ^*F*^ + const.) was controlled by using a potentiostat (CompactStat, Ivium). The PNL at the F|W interface was prepared as follows. The F (15 mL) was injected into the cell. The Pt-Ir ring was slowly lowered until ripples were observed on the surface of F, which indicated that the lower edge of the ring had touched the F surface. The ring was lowered a further 460−500 μm to position the height of the midplane of the ring diagonal to the surface. Then, 1 mM NaCl H_2_O solution (15 mL) was gently added on F to form the F|W interface. In the following experiments, the interfacial shear storage moduli *G*′ and loss moduli *G*″ did not change when the ring height was shifted by ± 300 μm. After the F|W interface was formed, *E*_F_^W^ = −0.6, −0.3, 0, +0.3, and +0.6 V were applied and then the BSA solution (pH 7.4, 15 mL) was slowly added to the W to start the formation of the PNL on the interface. The bulk concentration of BSA in the W_7.4_ was 0.5 mg/mL. The time dependence of *G*′, *G*″, and tan δ ( = *G*″/*G*′) of the PNL at the F|W interface was measured at a strain of γ = 1% and an angular frequency of ω = 1 Hz for 1 h, which is the saturation time of the BSA adsorption estimated from the interfacial tension measurements (See S1, Figure S1−2). 100% strain corresponds to the ring rotation over the same distance as that between the ring and outer wall in the radial direction.^51^ After 1 h, an amplitude sweep at 1 Hz was performed with a strain γ range of 0.1−200% (at *E*_F_^W^ = 0, +0.3, +0.6 V) and 0.1−1000% (*E*_F_^W^ = −0.3 V) to verify the strain resistance of the PNL at each *E*_F_^W^.

**Figure 2:**
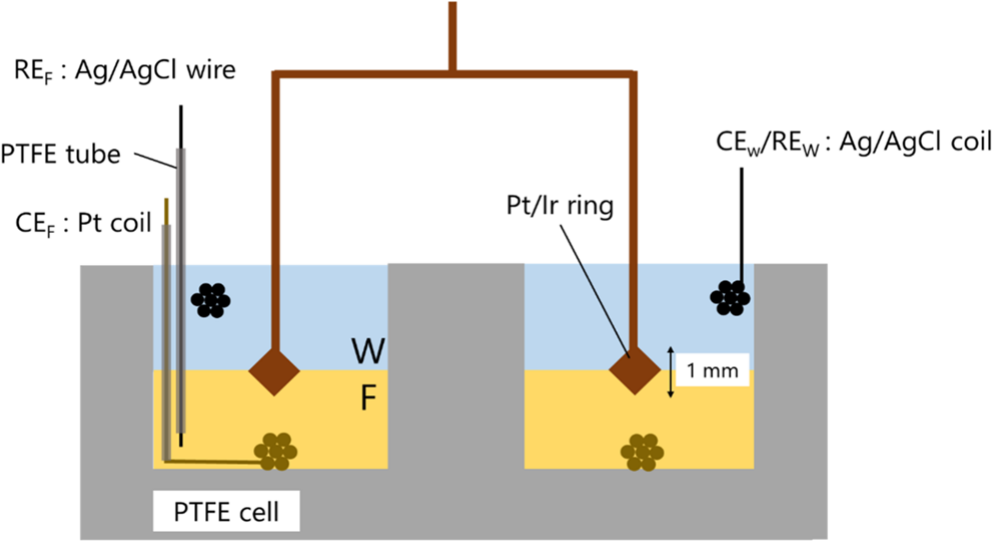
Schematic cross-section of IRM cell in a double wall-ring geometry. The electrodes for the F side are covered with PTFE tubes in W. The Pt-Ir ring wire was placed at the F|W interface. The cross-section of the ring was square with a vertical diagonal length of 1 mm.

## 3 Results and Discussion

### 3.1 Neutron Reflectometry

To analyze the amount of BSA adsorbed on the electrochemical F|W_7.4_ interface, *A*_BSA_ at the F|W_7.4_ interface was measured at each *E*_F_^W^ (Figure 3, red solid circles). The reflectivity data are shown in Figure S4−1. *E*_F_^W^ = 0 V should be close to the uncharged condition judging from the potential of zero charge (pzc) for the case without BSA, +0.05 V.^33^ The *A*_BSA_ at *E*_F_^W^ = 0 V was the same within the errors as the case using F without IL (Figure 3, a black plot shown at *E*_F_^W^ = 0 V). This illustrates that *A*_BSA_ is not affected with or without IL on the F side of the interface when the interface is not charged. With some degree of variability present in the data, the *A*_BSA_ with W_7.4_ slightly increased with decreasing *E*_F_^W^, suggesting that the BSA amount at the F|W interface increases at more negative potentials. This tendency was also observed in the interfacial tension measurements shown in Figure S1−2. At the BSA-free F|W interface, *E*_F_^W^ = 0.05 V is the pzc.^33^ Considering the composition of the electric double layer at the F|W interface,^33^ THTDP^+^ (C_4_C_4_N^−^) ions are more accumulated on the F side of the interface at *E*_F_^W^ < 0 V (*E*_F_^W^ > 0 V). At *E*_F_^W^ < 0 V, the electrostatic interaction between THTDP^+^ and BSA, which is negatively charged at pH 7.4, could increase the adsorption of BSA at the interface. This is similar to the electrodeposition of lysozyme at an O|W interface accelerated by the electrostatic interaction of positively charged lysozyme and anions in O.^52^ At *E*_F_^W^ > 0 V, although the electrostatic repulsion between C_4_C_4_N^−^ and BSA could decrease the adsorption of BSA at the interface, BSA was still adsorbed to the negatively charged interface to form the PNL as in a previous study where the positively charged part of BSA was adsorbed on a negatively charged silica surface.^53^ To further investigate the electrostatic interaction between BSA and the IL ions on the F side of the interface, we also performed NR at the F|W_2.6_ interface, using a pH 2.6 buffer where BSA is positively charged. The NR results at the F|W_2.6_ interface (Figure S5−3) were analyzed similarly to those at pH 7.4. The *A*_BSA_ of the PNL at the F (with IL)|W_2.6_ interface is shown in Figure S5−4. Opposite to the W_7.4_ case, with increasing *E*_F_^W^, the *A*_BSA_ values weakly increased, and therefore the BSA amount at the interface increased. This means that the Coulombic interaction behavior between IL ions and BSA at the F|W interface observed at pH 7.4 still holds at pH 2.6; the electrostatic repulsion (attraction) from THTDP^+^ (C_4_C_4_N^−^) at *E*_F_^W^ < 0 V (> 0 V) could decrease (increase) the adsorption of positively charged BSA at the interface. The slope sign change in the *A*_BSA_ vs *E*_F_^W^ plots at pH 2.6 indicates that the BSA amount in the PNL is controllable either by applying *E*_F_^W^ or by changing pH. It should be noted, however, that the *E*_F_^W^ dependence of the BSA adsorption amount, revealed using NR and interfacial tension measurements, was minimal, which is in stark contrast to a dramatic change in the viscoelasticity shown below.

**Figure 3:**
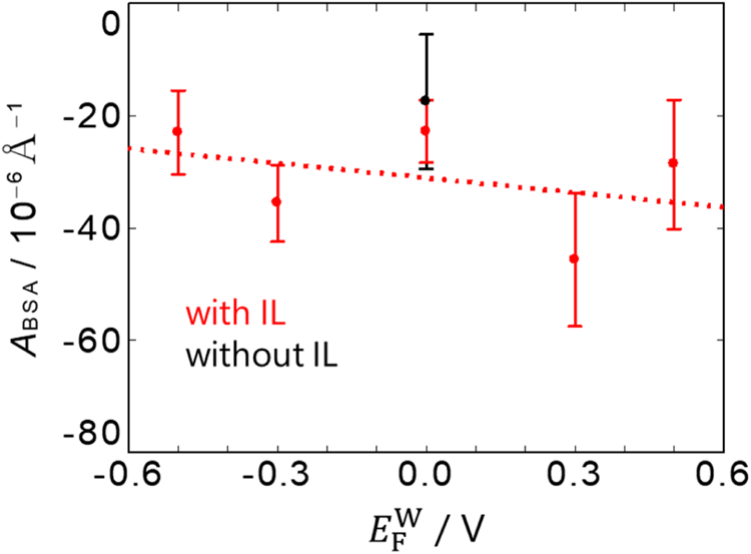
Potential dependence of *A*_BSA_ of the PNL at the interface between W_7.4_ and F with IL (red). The data for the neat F case without IL, and therefore without external potential control, is shown at *E*_F_^W^ = 0 V (black) for comparison. The error bars are the standard errors of fitting results. The red dotted line is from the least-squares fitting for the data with IL.

### 3.2 Interfacial Rheological Measurements

To analyze how the phase boundary potential affects the viscoelasticity of the PNL, we performed the IRM at the electrochemical F|W_7.4_ interface. The *G*′ (storage modulus) and *G*″ (loss modulus) obtained from IRM provide insight into the mechanical characteristics of the PNL at the interface. *G*′ reflects the elastic nature, likely originating from reversible intermolecular interactions between BSA molecules, such as hydrogen bonding, hydrophobic association, or electrostatically mediated clustering. *G*″, in contrast, is associated with dissipative processes, including rearrangement of protein molecules or partial unfolding of loosely bound protein dangling ends or interfacial viscosity. These mechanical parameters are highly relevant to cell culture applications. Recent studies in mechanobiology have revealed that not only *G*′ but also *G*″ plays a critical role in regulating cell behavior on viscoelastic substrates. For instance, the “molecular clutch model” suggests that viscous dissipation influences focal adhesion dynamics and actin flow in addition to stiffness.^54,55^ Cooper et al. also reported that changes in *G*″, with constant *G*′, modulate cell spreading and cytoskeletal organization. Therefore, the ability to electrochemically and reversibly tune both *G*′ and *G*″ at a liquid|liquid interface offers a promising approach to investigating dynamic mechanosensing in cells.^56,57^

We first examined the effect of IL addition to F on the viscoelasticity of PNL at the F|W interface. The time evolution of interfacial shear storage and loss moduli, *G*′ and *G*″, respectively, for 1 h is shown in Figure S5−1. For both the cases in the presence and absence of IL, one can see that *G*′ and *G*″ increase with increasing time, keeping *G*′ > *G*″, which means the formed PNL has a gel-like nature. The difference between the two cases is clearly discernible in Figure S5−1. The two moduli steeply rise for the case without IL on the order of 100 s, contrasting with a 10 times slower PNL formation when F contained IL. The slowdown in the presence of IL can be explained by the fact that the F side of the F|W interface is covered by an IL ion-rich layer when F contains IL, even without applying external potential, which was unveiled in our previous NR study.^33^ Such accumulation of IL ions leads to the slow formation of the interfacial structure,^58^ resulting in the slow rise in the two moduli in the presence of IL, as shown in Figure S5−1. This indicates that the IL, even though it is seemingly an additive in F with a low concentration (2.5 mM), has a strong impact not only on the interfacial structure but also on the viscoelasticity of PNL on the interface. Then we investigated the time evolution of the two moduli in the IL-added case for a longer time (>10 h), the results of which are shown in Figure S5−2. Note that in this case, we applied *E*_F_^W^ = 0 V, which is close to the uncharged condition without potential control. Figure S5−2 shows both *G*′ and *G*″ evolve on the order of 10^3^ with a steeper increase in the *G*′, indicating that the PNL gradually exhibits a more elastic-dominant response on this time scale (11 h). In the following, we examine the effect of the phase boundary potential on the PNL. It is noted that *G*′ and *G*″ did not reach equilibrium at 1 h. Figure 4 shows the time evolution of *G*′ and *G*″ during 1 h after the BSA solution was injected at *E*_F_^W^ = −0.6, 0, +0.6 V. As *E*_F_^W^ decreased, *G*′ and *G*″ increased more steeply. Considering that the time evolution of the surface pressure (Figure S1−2) was not significantly different regardless of *E*_F_^W^, IRM indicated that at lower *E*_F_^W^ BSA was more denatured and formed more intermolecular bonds. Figure 5a shows the *G*′ and *G*″ 1 h after the injection of the BSA solution at *E*_F_^W^ = −0.6, −0.3, 0, +0.3, +0.6 V. As *E*_F_^W^ decreased, *G*′ significantly increased, whereas *G*″ slightly increased, which shows that the PNL was more elastic (lower tan δ) at *E*_F_^W^ = −0.6 V (on the positively charged F surface), and was more viscoelastic (higher tan δ) at *E*_F_^W^ = 0.6 V (on the negatively charged F surface). Controlling *E*_F_^W^ had a significant effect on the viscoelasticity of the PNL at the F|W_7.4_ interface (Figure 5a) unlike that in the amount of BSA in the PNL (Figures 3, S1−2b, S4−4). At *E*_F_^W^ < 0 V, IL cations are more accumulated at the F side of the interface.^33^ Charged proteins at the electrochemical O|W interface were reported to form complexes with organic ions in O that have the counter charge.^52,59–62^ Similarly, in the present study, the complex formation of BSA, which is negatively charged, with IL cations is likely to accelerate the denaturation of BSA depending on *E*_F_^W^, which strengthened the intermolecular and intramolecular bonds of BSA in the PNL. In addition to the denaturation of BSA, the viscosity of ILs at the interface,^63–74^ including at the liquid|liquid interface,^58,70,75^ was reported to be much higher than that in the IL bulk because of spontaneously formed well-ordered ionic multilayers at the interface. Our previous study on the ionic compositions at the F|W interface revealed that IL ions are accumulated at the interface, especially at *E*_F_^W^ < 0 V up to 400 times higher concentrations than those in the bulk,^33^ forming an IL-like environment at the interface. This implies that at *E*_F_^W^ < 0 V, well-ordered IL-rich layers are formed at the interface, contributing to increased stiffness and more elastic interfacial behavior of the PNL.

**Figure 4:**
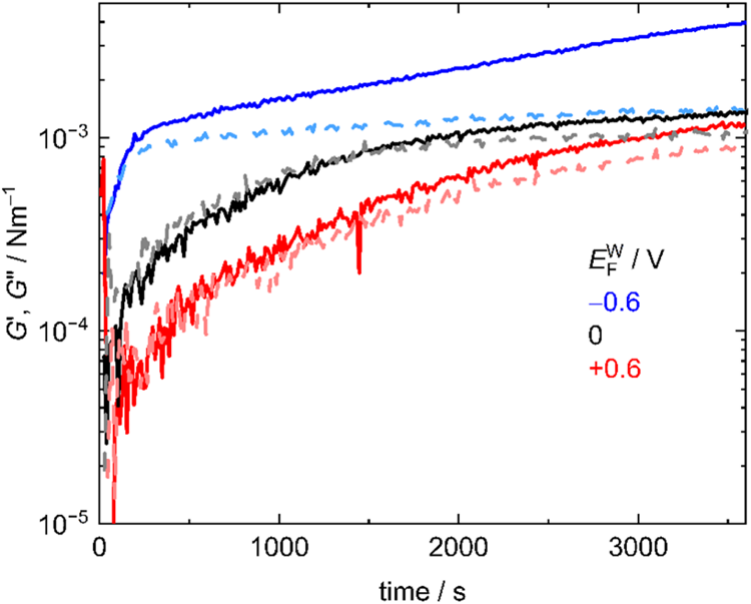
Time evolution of interfacial shear storage and loss modulus *G*′ (solid lines) and *G*″ (dashed lines) at *E*_F_^W^ = − 0.6 (blue), 0 (black), + 0.6 V (red) at the F|W interface after *t* = 0 when the BSA solution was injected. *G*′ and *G*″ were measured with a strain of γ = 1% and an angular frequency of ω = 1 Hz. Those at *E*_F_^W^ = −0.3 and +0.3 V are shown in Figure S5−4 and 6, respectively.

**Figure 5:**
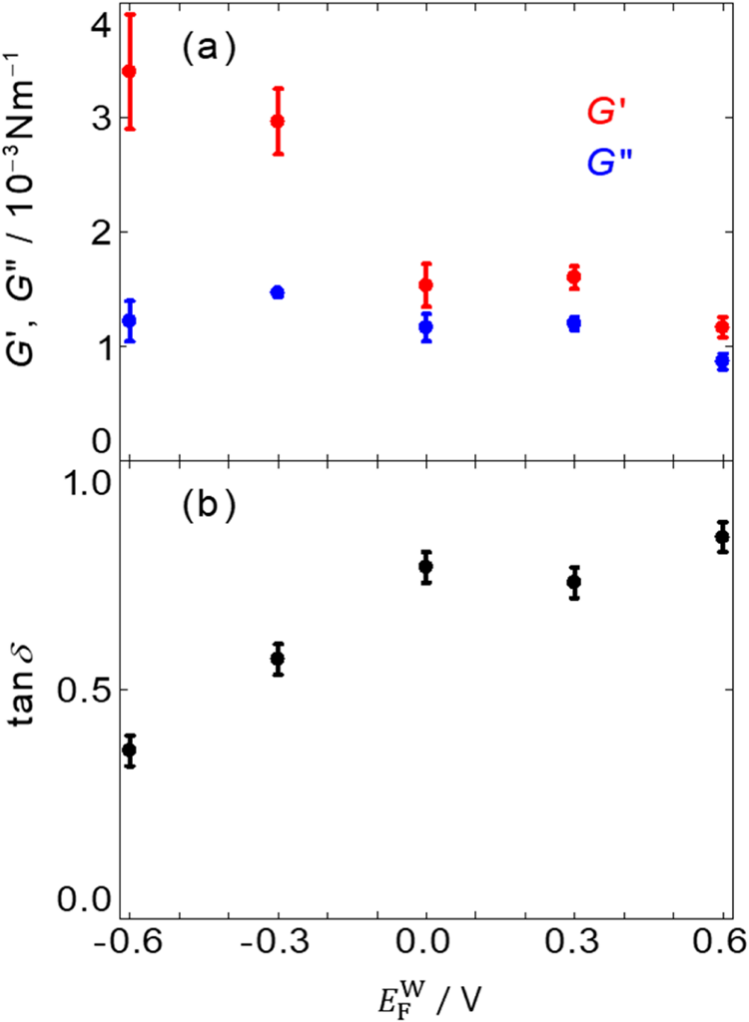
Potential dependence of (a) interfacial shear storage and loss modulus, *G*′ (red) and *G*″ (blue), and (b) tan δ (= *G*″/*G*′, black) at the F|W interface 1 h after the BSA solution was injected. *G*′ and *G*″ were measured with a strain of γ = 1% and an angular frequency of ω = 1 Hz. The error bars are the standard errors from three experiments at each *E*_F_^W^ shown in Figure S5−3–7.

At *E*_F_^W^ > 0 V, PNL showed less elasticity than that at *E*_F_^W^ < 0 V. BSA at the interface is likely to be less denatured because the F side of the interface was covered by relatively hydrophilic sulfonyl groups of C_4_C_4_N^−^ that orient their perfluorobutyl groups to the F bulk, according to the compositional analysis of the F|W interface^33^ and the orientational one of the IL ions at the IL|W interface.^76^ These factors could inhibit the formation of intermolecular bonds between neighboring BSA and make the PNL less elastic at *E*_F_^W^ > 0 V. Figure 5b shows the loss tangent of the PNL, tan δ (= *G*″/*G*′), at each *E*_F_^W^. As *E*_F_^W^ increased, tan δ increased from 0.4 at *E*_F_^W^ = −0.6 V to 0.8 at *E*_F_^W^ = 0.6 V, which also indicated that the PNL is more elastic (lower tan δ) at *E*_F_^W^ = −0.6 V and more viscous (higher tan δ) at *E*_F_^W^ = 0.6 V. These results show that *E*_F_^W^ can switch the viscoelasticity of the PNL at the F|W interface. Figure 6 shows *G*′ and *G*″ profiles as a function of strain γ which were measured at 1 h after the BSA solution was injected. The points in Figure 6 are the yield points where *G*′ and *G*″ profiles cross over (*G*′ = *G*″), γ_YP_, which is a measure of how resistant the PNL is to strain. As *E*_F_^W^ decreased from *E*_F_^W^ = +0.6 to −0.6 V, the γ_YP_ increased, indicating that the structure of PNL at *E*_F_^W^ < 0 is much more strain-resistant. Figure 7 shows the *E*_F_^W^ dependence of γ_YP_. In Figure 7, γ_YP_ at *E*_F_^W^ = −0.6 V reached 347%, which came from a peculiar behavior in the decaying parts of *G*′ and *G*″ profiles, where the former showed plateaus around 100% strain and then crossed over at greater strain (Figure S5−8a,b). These peculiar behaviors in the decaying parts were observed in all experiments (3 out of 3 times) at *E*_F_^W^ = −0.6 V (Figure S5−8), sometimes observed (2 out of 5 times) at *E*_F_^W^ = −0.3 V (Figure S5−9b). Similar behavior was also observed in Figure S5−9c, with the decay in *G*′ being smaller at 100%. In contrast, those behaviors were not observed (0 out of 3 times) at *E*_F_^W^ = 0, +0.3, and +0.6 V (Figures S5−10–12). In Figure 7, the γ_YP_ at *E*_F_^W^ = −0.3 V was not taken into account when the peculiar behavior was observed. The peculiar behavior of the *G*′ and *G*″ at *E*_F_^W^ = −0.6 and −0.3 V might be because the reduction in *G*′ decreased due to the reformation of the PNL structure and new interactions between BSA molecules at large strains around γ = 100%. At *E*_F_^W^ = 0, +0.3, and +0.6 V, the plateau was not observed probably because the number of BSA in PNL was smaller and BSA was less denatured, making it harder to reform the PNL structure and new interactions between BSA molecules. The results in Figures 5–7 indicate that PNLs at *E*_F_^W^ < 0 V have both the high elasticity and the high strain resistance without any chemical treatments to PNL.

**Figure 6:**
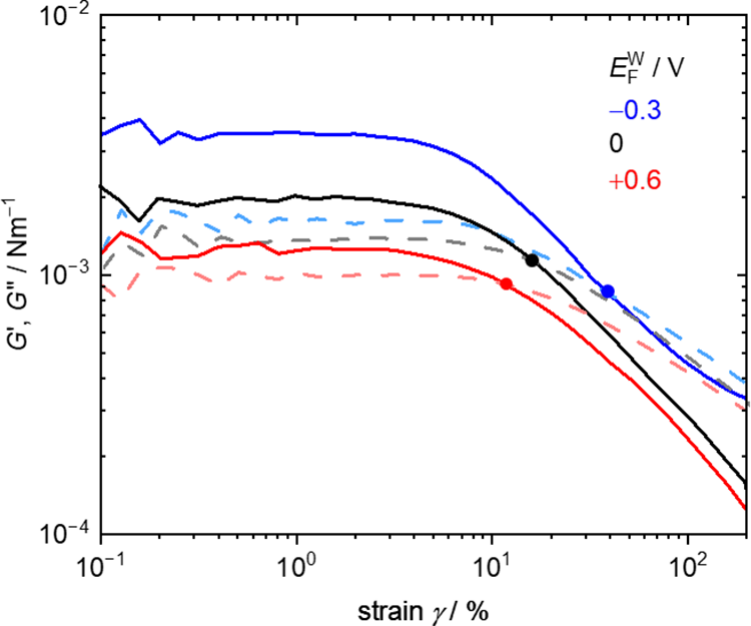
Strain γ sweep of interfacial shear storage *G*′ (solid lines) and loss modulus *G*″ (dashed lines) at *E*_F_^W^ = − 0.3 (blue), 0 (black), +0.6 V (red) at the F|W interface at 1 h after the BSA solution was injected. The points are the yield point where *G*′ and *G*″ profiles cross over (*G′* = *G*″), γ_YP_. These profiles are the ones whose γ_YP_ are the closest to the average of γ_YP_ out of the three profiles at *E*_F_^W^ = −0.3, 0, and +0.6 V shown in Figure S5−9, 10, and 12, respectively. Those at *E*_F_^W^ = −0.6 and +0.3 V are shown in Figure S5−8 and 11, respectively.

**Figure 7:**
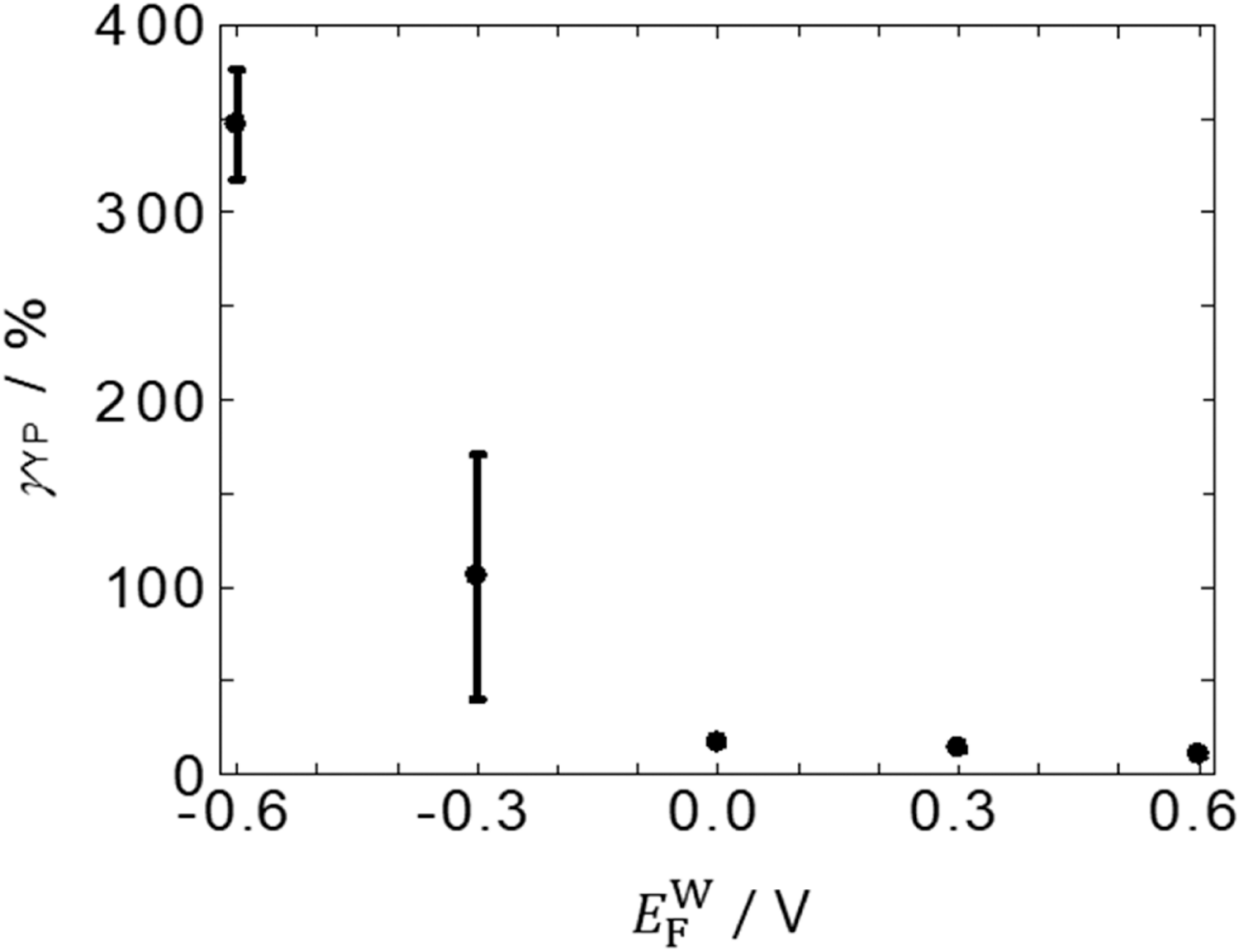
γ_YP_ of the PNL at the F|W interface.

The PNL at the liquid|liquid interface was reported to recover after cracking the PNL.^18^ The following experiments were carried out to investigate the potential dependence of the recovery speed of the cracked PNL. After the PNL had been formed for 1 h, it was cracked by applying a 1000% deformation at *E*_F_^W^ = −0.6, 0, +0.6 V and then the time sweep was performed to measure the recovery time for reaching the values of *G*′ and *G*″ before cracking (Figure S5−13). The recovery time was 10 s at *E*_F_^W^ = −0.6 V, 350 s at *E*_F_^W^ = 0 V, and 600 s at *E*_F_^W^ = 0.6 V; as *E*_F_^W^ decreased, the recovery time was shortened. The electrostatic attraction is likely to accelerate the adsorption of BSA and recovery of the PNL at the F|W interface. This tendency agrees with the case at the silica|W interface where the adsorption of positively charged BSA at pH > 5 is faster than that of negatively charged BSA at pH < 5.^77^ The recovery time at *E*_F_^W^ = 0 V, 350 s, is comparable to that at the IL|W interface measured by using high-speed AFM, 300−500 s.^18^ Although F is a diluted solution of IL, the recovery time at the F|W and IL|W interfaces without externally controlling the phase boundary potential difference was close. This also supports that the composition of the F side of the F|W interface is IL-like.^33^

The switching effect of *E*_F_^W^ on *G*′ and *G*″ of the PNL was investigated to control the structure and mechanical property of PNL. Figure 8 shows the profiles of *G*′ and *G*″ when the *E*_F_^W^ was switched between *E*_F_^W^ = +0.3 V and *E*_F_^W^ = −0.6 V. *G*′ and *G*″ were reversible against the *E*_F_^W^ switch, implying that the mechanical interaction of PNL and cells on the electrochemical F|W interface is actively switchable. The transition of *G*′ and *G*″ is different between *E*_F_^W^ = +0.3 to −0.6 V and *E*_F_^W^ = −0.6 to +0.3 V. This indicates that the structural changes of the PNL induced by switching *E*_F_^W^ have various processes such as the adsorption and desorption of BSA, and the rearrangement of the adsorption part and the inter and intramolecular bonds of BSA. The fact that *G*″ responds more rapidly to potential switching than *G*′ may suggest that dissipative processes such as rearrangement of protein molecules or partial unfolding of loosely bound protein dangling ends occur quickly, whereas the development of a more elastic network (reflected in *G*′) involves slower maturation of intermolecular interactions. This difference implies that multiple, time scale-dependent processes contribute to the viscoelastic modulation of the PNL.

**Figure 8:**
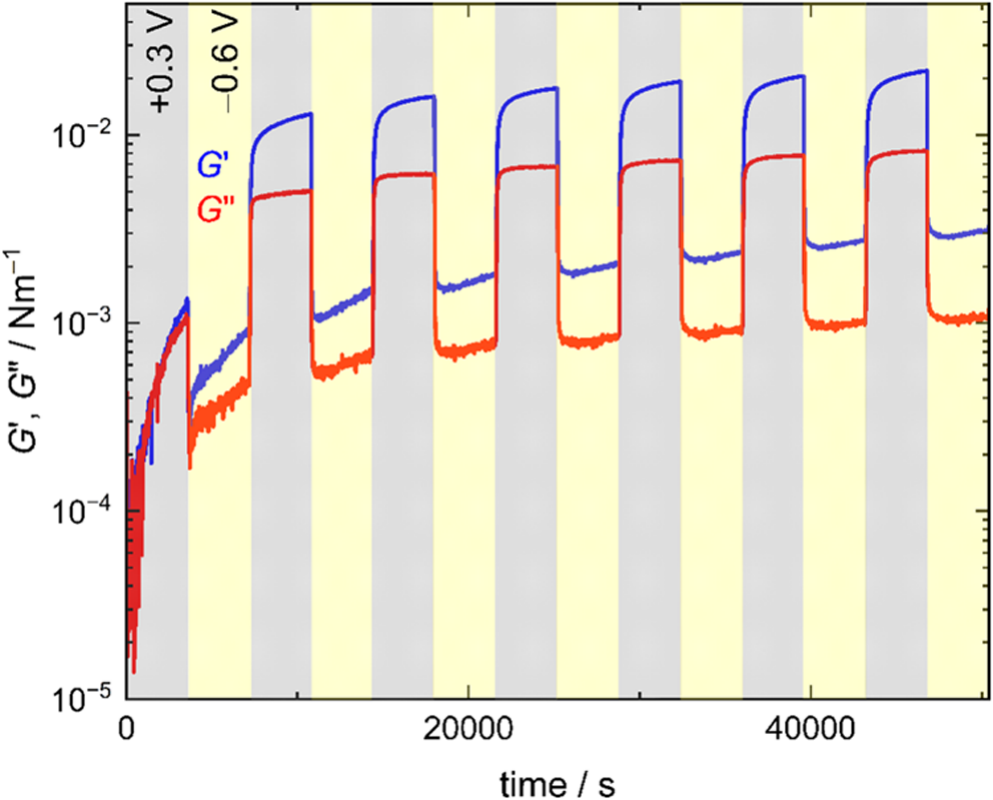
Time courses of *G*′ (blue) and *G*″ (green) of the PNL against multiple potential switches between at *E*_F_^W^ = +0.3 (gray) and −0.6 V (yellow) at every 3600 s.

## 4 Conclusions

We successfully analyzed the PNL structure at the F|W interface under the condition of *E*_F_^W^ by using NR and IRM. Although the NR results showed that *E*_F_^W^ has minimal effect on the adsorption amount of BSA in the PNL at the electrochemical F|W interface, IRM unveiled that the in-plane structure was strengthened at *E*_F_^W^ < 0 V, and was reversibly switchable by applying *E*_F_^W^. The present proof-of-concept study demonstrates that the potential control is an interface-specific method to diversify and switch the PNL structure reversibly. This method with interface-specificity and reversibility is in stark contrast to previously reported ones that change the hydrophobic liquids^4,14,18,26,43,78,79^ or add reagents in W.^5,13,14,26,34^ The reversible structural control of the PNL would enable us to perform real-time observation of cells reacting to environmental changes at liquid|liquid interfaces.

## Supplementary Material



## Notes

Interfacial tension measurements at the F|W interface; NR at the liquid|air interface; interfacial roughness of the F|W interface evaluated using the capillary wave theory; and NR and interfacial rheological measurements at the F|W interface (PDF)
