# Supplementary material for: Potential-Switchable Viscoelasticity of Protein Nanolayers at a Liquid/Liquid Interface
Source: Langmuir. 2025 Jul 1;41(27):17973–81. doi: 10.1021/acs.langmuir.5c01819 (PMC12400409; doi:10.1021/acs.langmuir.5c01819)
Supplement: Supplementary file 1 [file la5c01819_si_001.pdf]

Supporting Information

Potential-switchable viscoelasticity of protein nanolayers at a liquid/liquid interface

Kosuke Ishii[1], Takeshi Ueki[2,3,*], Jun Nakanishi[2,4,5], Kazuhiro Akutsu-Suyama[6], Norifumi L. Yamada[7], Yuko Yokoyama[1], Tetsuo Sakka[1], and Naoya Nishi[1,*]

[1]*Department of Energy and Hydrocarbon Chemistry, Kyoto University, Kyoto 615-8510, Japan*

[2]*Research Center for Macromolecules & Biomaterials, National Institute for Materials Science (NIMS), 1-1 Namiki, Tsukuba, Ibaraki 305-0044, Japan*

[3]*Graduate School of Life Science, Hokkaido University, Kita 10, Nishi 8, Kita-ku Sapporo 060-0810, Japan*

[4]*Graduate School of Advanced Science and Engineering, Waseda University, 3-4-1 Okubo, Tokyo, Shinjuku-ku 169-8555, Japan*

[5]*Graduate School of Advanced Engineering, Tokyo University of Science, 6-3-1 Niijuku Tokyo, Katsushika-ku 125-8585, Japan*

[6]*Neutron Science and Technology Center, Comprehensive Research Organization for Science, and Society (CROSS), Tokai, Naka 319-1106, Ibaraki, Japan*

[7]*Neutron Science Laboratory, Center for Integrative Quantum Beam Science, High Energy Accelerator Research Organization, Tokai, Naka 319-1106*

**Corresponding author: Naoya Nishi: nishi.naoya.7e@kyoto-u.ac.jp*

Takeshi Ueki: UEKI.Takeshi@nims.go.jp

S1 Interfacial tension measurements

We measured the time dependence of the interfacial tension at the fluorous solvent (F)|water (W) interface with a protein nanolayer (PNL) using a pendant drop method. The setup was the same as our previous study on the electric double layer at the F|W interface [1] with a positive-feedback IR compensation (Fig.S1-1). The interfacial tension was evaluated from the contour of a pendant drop of F in W as described elsewhere.[2,3] The time dependences of the interfacial tension were measured as follows. A droplet of F was formed in a 1 mM NaCl D_2O solution (15 ml). Then, a BSA solution (see Section 1.1) was added to form the PNL. The first picture of the pendant drop was taken within 10 minutes after the injection of the BSA solution. Fig.S1-2a shows the time dependences of the interfacial tension at E_F^W= –0.3, 0, +0.3 V. The interfacial tension decreased to 18 mN m^{-1} at E_F^W= +0.3 and 0 V, and 13 mN m^{-1} at E_F^W= –0.3 V in 1 hour. The surface pressure at the F|W interface (Fig.S1-2b) was saturated to 18 mN m^{-1} at E_F^W= –0.3 and 0 V, and 16 mN m^{-1} at E_F^W= +0.3 V in 1 hour, which implies that the absorbed amount of BSA to the interface at E_F^W= –0.3 and 0 V was larger than that at E_F^W= +0.3 V. The adsorption of BSA at the F|W interface was estimated to be saturated in 1 hour.

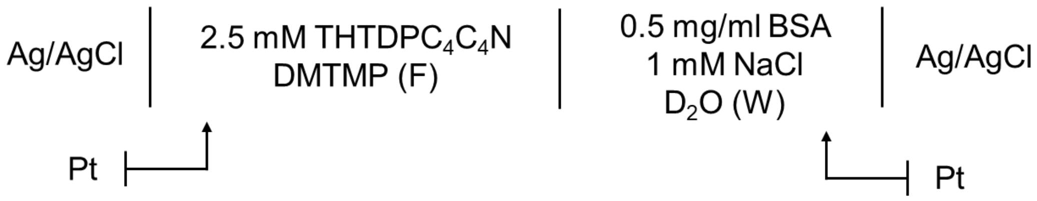

Fig.S1-1 Electrochemical system of the setup for electrocapillary measurement at the interface between 2.5 mM THTDPC$_4$C$_4$N DMTMP solution and D_2O solution containing 0.5 mg/ml BSA and 1 mM NaCl.

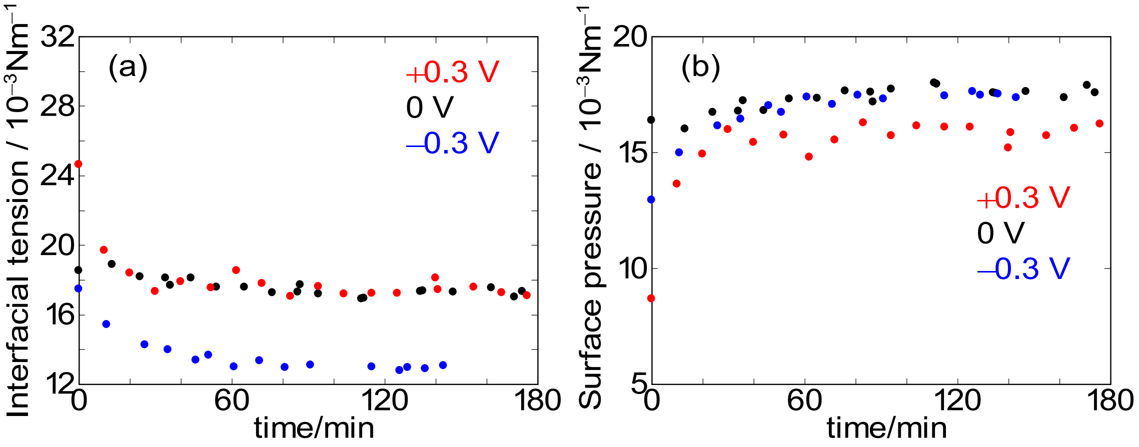

Fig.S1-2 Time dependence of (a) the interfacial tension and (b) the surface pressure at the F|W interface. The surface pressure in (b) was evaluated from the interfacial tension difference between the BSA-adsorbed and BSA-free F|W interfaces. [1] The time = 0 is when the first photograph of the droplet was taken.

S2 Neutron reflectometry at the liquid|air interface

We performed neutron reflectometry (NR) at the F|air and W|air interface to determine the bulk scattering length density (SLD) of F and W using another cell [6]. The q range was 0.01–0.04 $Å^{-1}$ (the incident angle was 0.4°). The other conditions are the same as for NR at the liquid-liquid interface [6]. The SLD of W and F was determined to $\rho_W = 6.16\times10^{-6}$ $Å^{-2}$, and $\rho_F = 3.13\times10^{-6}$ $Å^{-2}$ from the critical angle for total reflection in the reflectivity profiles at the F|air and W|air interface.

S3 Capillary wave theory

The surface roughness σ_A at the layer boundaries between W-L and L-F was calculated in the following using the capillary wave theory [4,5]. σ_i is expressed in the following equation SEq. 1 [4].

$$\sigma_i{}^2 = \frac{k_B T}{2\pi\gamma_i}\ln\left(\frac{q_{max}}{q_{min}}\right) \qquad \text{(SEq. 1)}$$

where k_B is the Boltzmann constant, T is the absolute temperature, 298 K, and γ_i is the interfacial tension measured using the electrocapillary measurement at each E_F^W (Fig.S1-2a). q_{min} is the smallest wavenumber determined by the size of the beam footprint (20 mm × 30 mm, equation SEq. 2).

$$q_{min} = \frac{2\pi}{L} \qquad \text{(SEq. 2)}$$

where L is the long side of the footprint (30 mm). The value of q_{max} is the maximum wavenumber determined by the size of the molecule D_{mol} (equation SEq. 3).

$$q_{max} = \frac{2\pi}{D_{mol}} \qquad \text{(SEq. 3)}$$

where D_{mol} was assumed to be the cubic root of the volume of BSA, 44 Å. We used σ_A = 5.0 Å as the average of σ_i; 5.6, 4.7, 4.7 Å at E_F^W= –0.3, 0, +0.3 V, respectively.

S4 Neutron reflectometry at the F|W interface

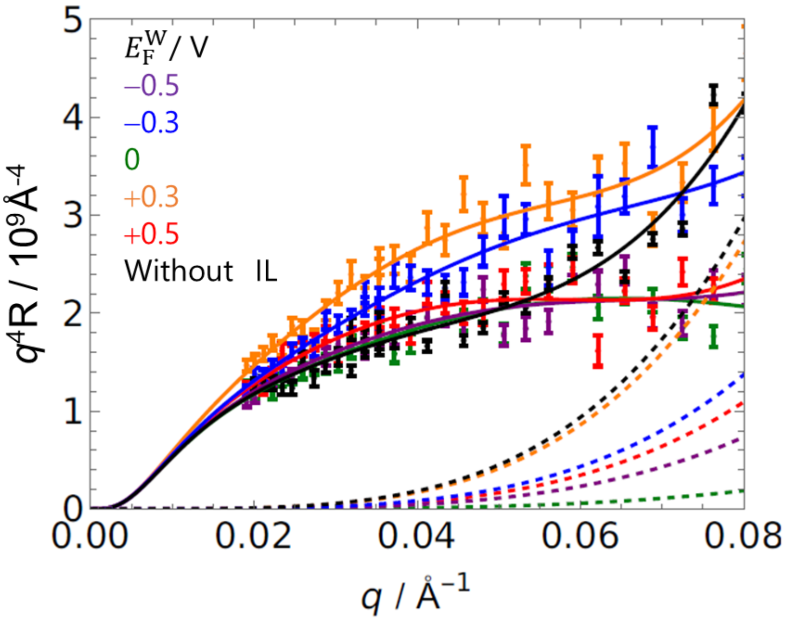

Fig.S4-1 Neutron reflectivity as a function of momentum transfer q of the PNL at the F (with IL)|$W_{7.4}$ interface at E_F^W = −0.5 (purple), −0.3 (blue), 0 (green), +0.3 (orange), and +0.5 V (red), and at the F (without IL)|$W_{7.4}$ interface (black). The solid lines are obtained from the fitting using a one-slab model **with fixing** $\sigma_{L\text{-}F}$. The dotted lines are the fitted background.

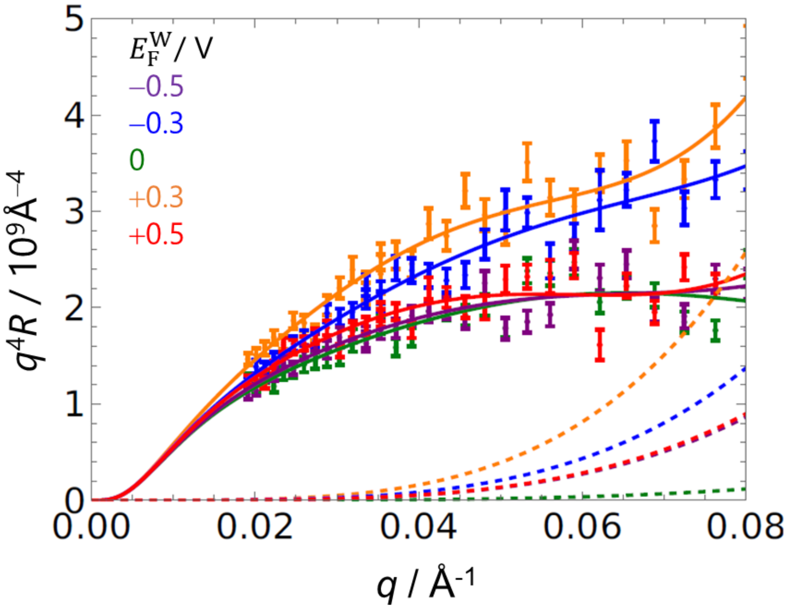

Fig.S4-2 Neutron reflectivity as a function of momentum transfer q of the PNL at the F|$W_{7.4}$ interface at E_F^W = −0.5 (purple), −0.3 (blue), 0 (green), +0.3 (orange), and +0.5 V (red). The solid lines are obtained from the fitting using a one-slab model **without fixing** $\sigma_{L\text{-}F}$. The dotted lines are the fitted background.

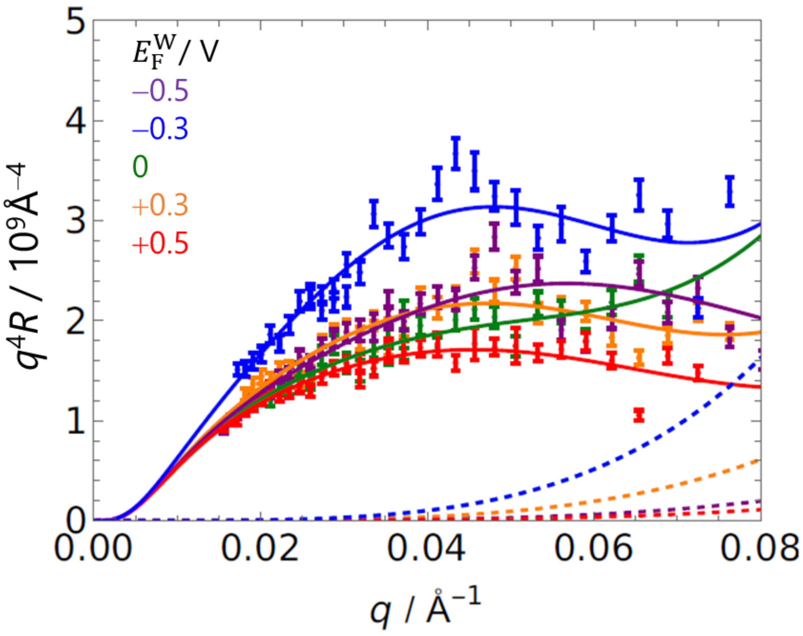

Fig.S4-3 Neutron reflectivity as a function of momentum transfer q of the PNL at the F|$W_{2.6}$ interface at E_F^W = −0.5 (purple), −0.3 (blue), 0 (green), +0.3 (orange), and +0.5 V (red). The solid lines are obtained from the fitting using a one-slab model **with fixing σ_{L-F}**. The dotted lines are the fitted background.

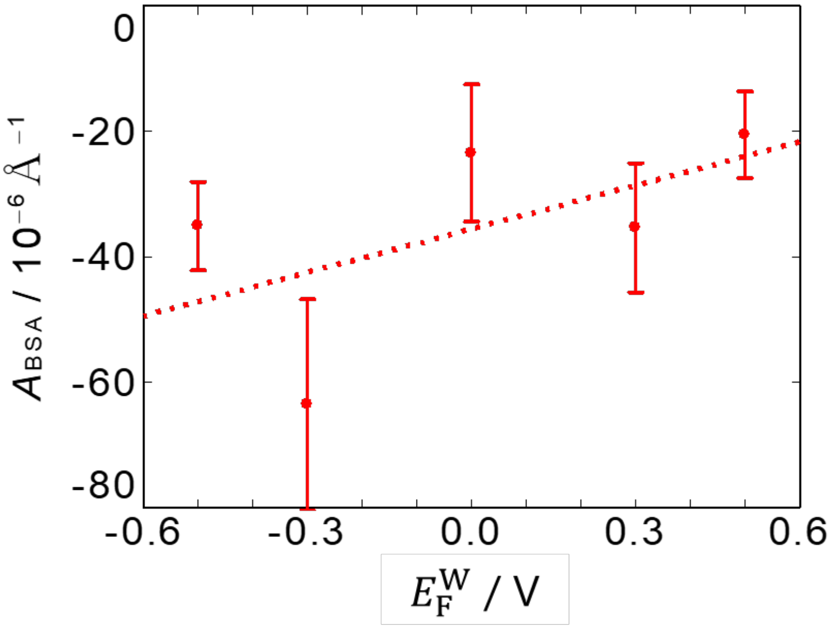

Fig.S4-4 A_{BSA} of the PNL at the F|$W_{2.6}$ interface as a function of E_F^W. The dotted line is obtained by linear fitting of the dots.

Table S4-1 Akaike Information Criterion (AICc); the fixed vs. non-fixed surface roughness $\sigma_{L\text{-}F}$.

	AICc[a)]	
E_F^W / V	fixed $\sigma_{L\text{-}F}$	non-fixed $\sigma_{L\text{-}F}$
−0.5	−108.2	−104.9
−0.3	−121.9	−118.7
0	−97.5	−94.1
+0.3	−116.7	−113.4
+0.5	−101.5	−98.1

a) A model with a smaller AICc is more likely than another.

Table S4-2 Fitted Parameters of the F|$W_{7.4}$ interface at various E_F^W without fixing $\sigma_{L\text{-}F}$.

E_F^W / V	A_{BSA} / 10^{-6} Å^{-1}	$\sigma_{L\text{-}F}$/Å
−0.5	−47	14
−0.3	−60	13
0	−19	0
+0.3	−43	0
+0.5	−26	0.4

S5 Interfacial rheological measurements

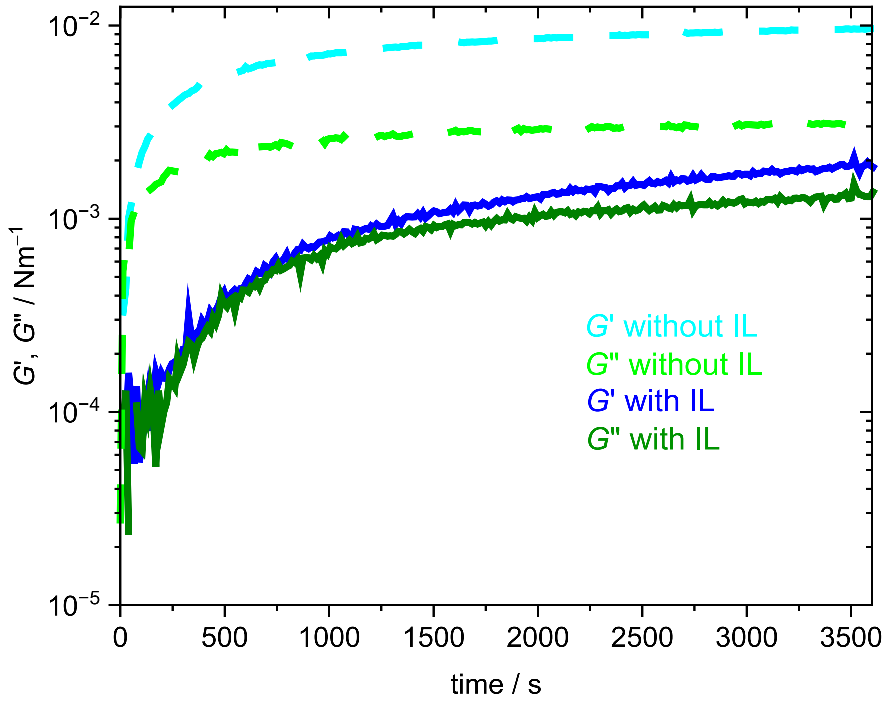

Fig.S5-1 *G′* and *G″* of the PNL at the W interface of F with/without IL as a function of time.

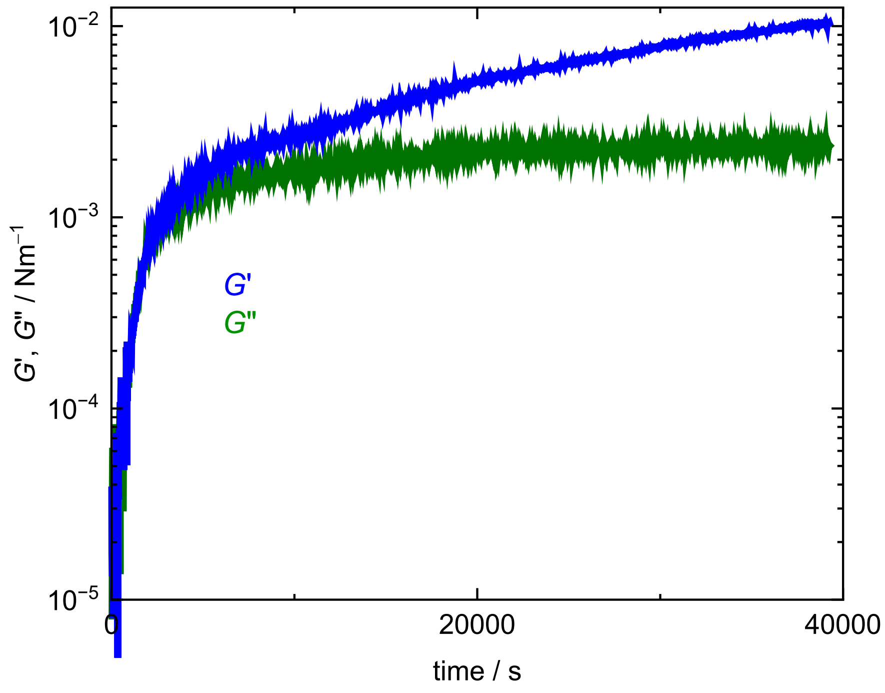

Fig.S5-2 *G′* and *G″* of the PNL formed in the addition of IL in F at $E_F^W = 0$ V as a function of time.

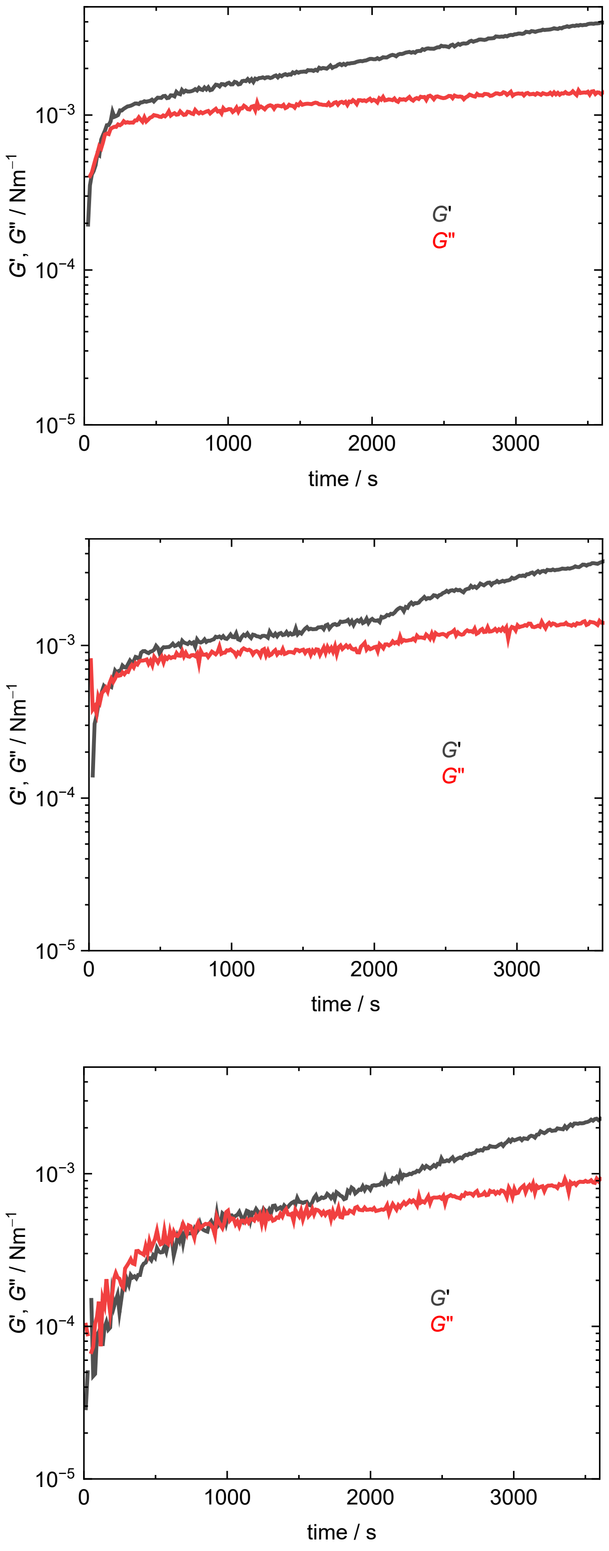

Fig.S5-3 Time evolution of G' (black) and G'' (red) at E_F^W = –0.6 V after the BSA solution was injected. The top one is shown in Fig.4.

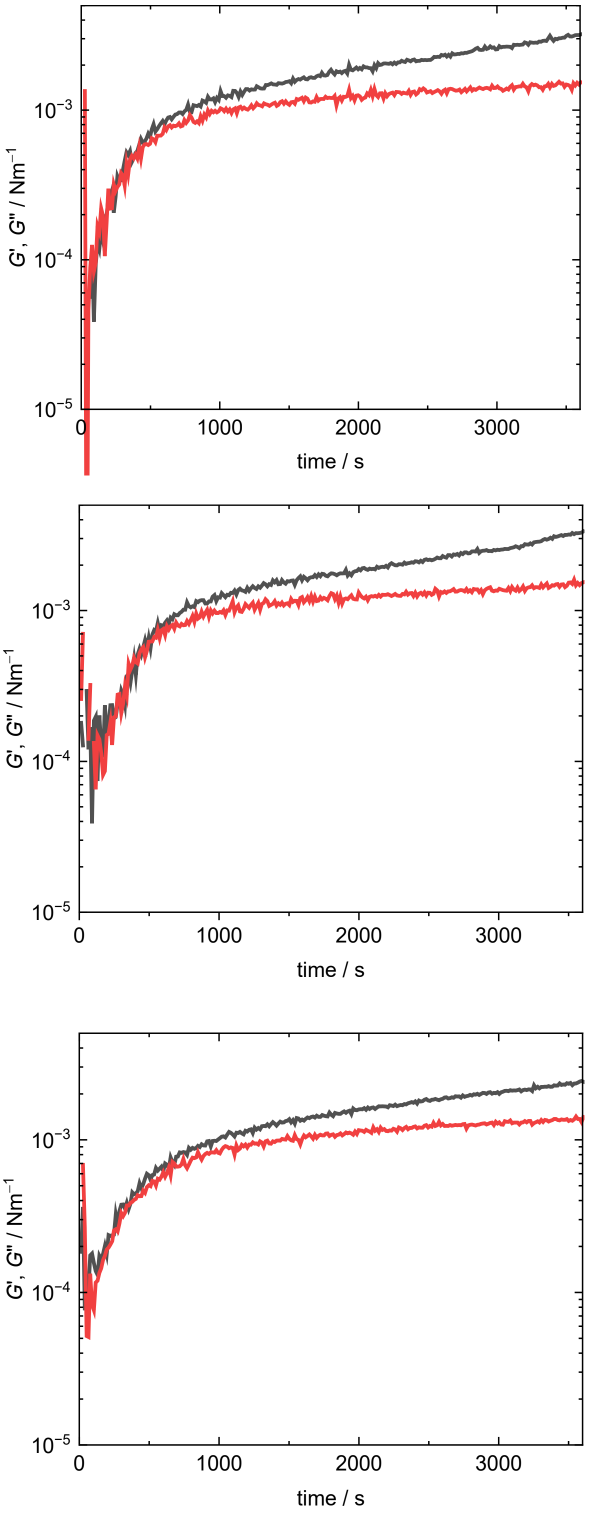

Fig.S5-4 Time evolution of G' (black) and G'' (red) at $E_{\mathrm{F}}^{\mathrm{W}}$ = –0.3 V after the BSA solution was injected.

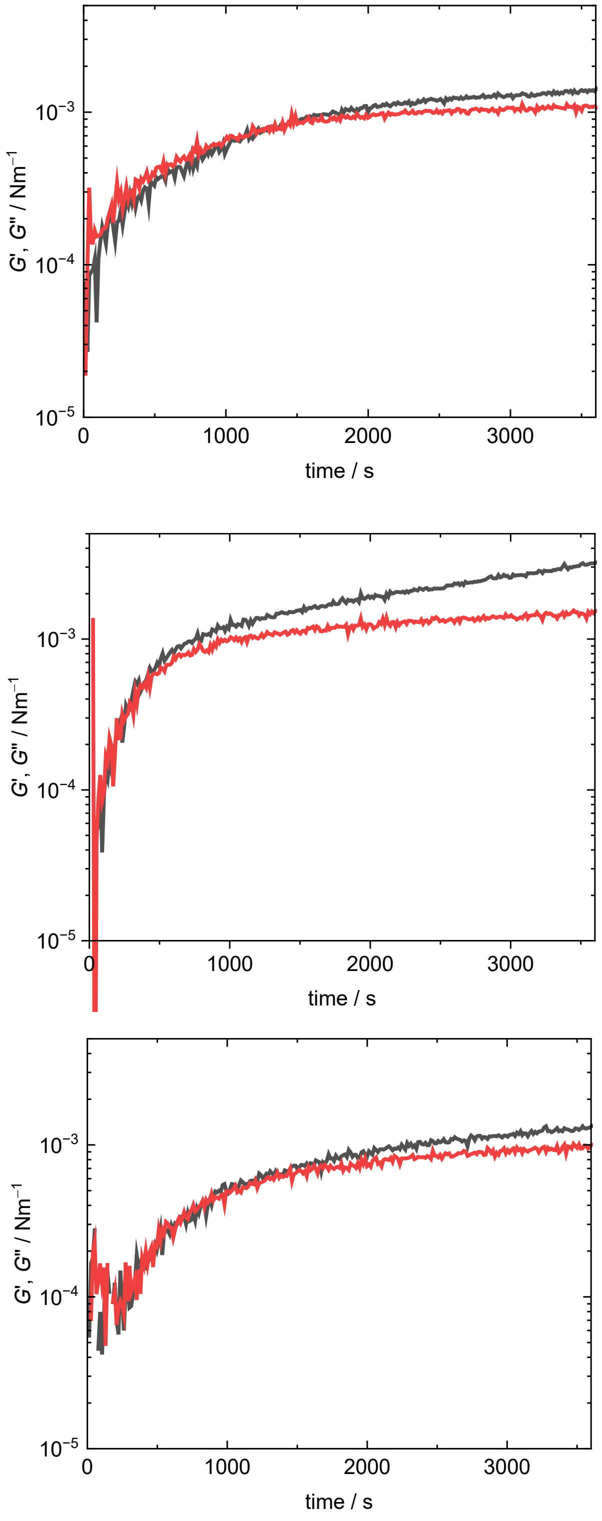

Fig.S5-5 Time evolution of G' (black) and G'' (red) at E_F^W = 0 V after the BSA solution was injected. The top one is shown in Fig.4.

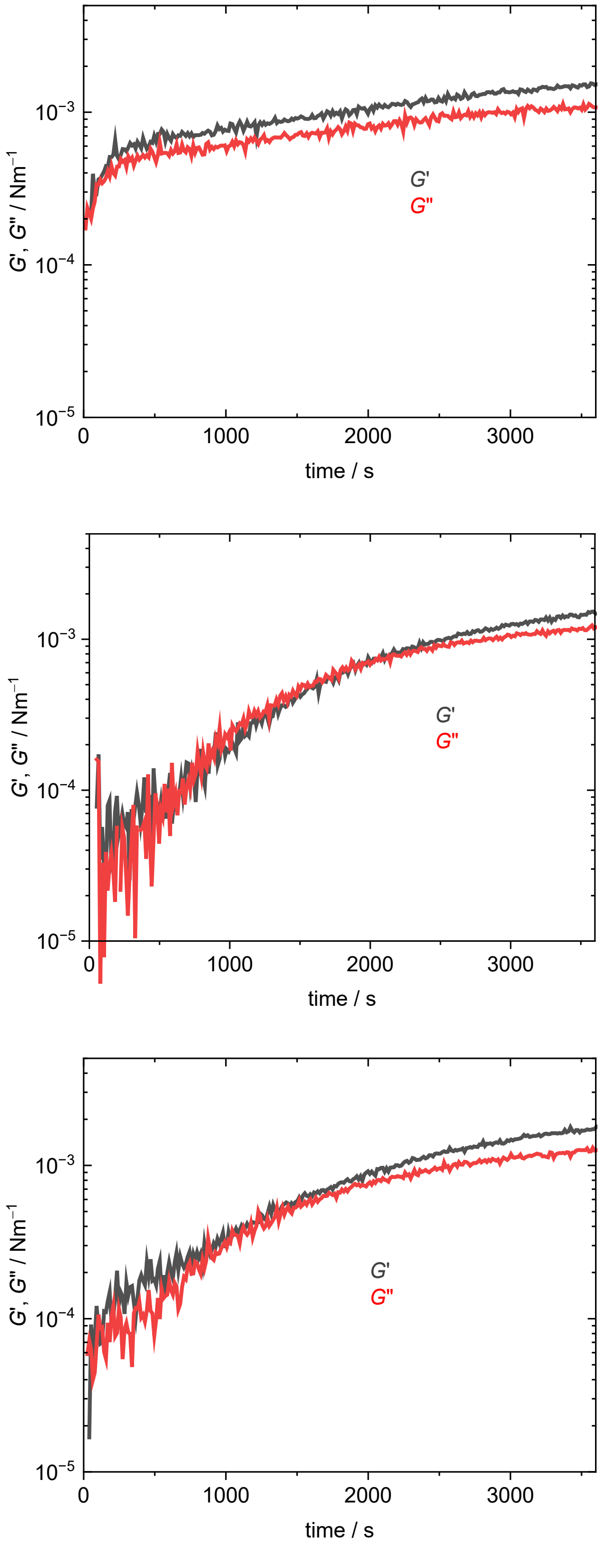

Fig.S5-6 Time evolution of G' (black) and G'' (red) at E_F^W = +0.3 V after the BSA solution was injected.

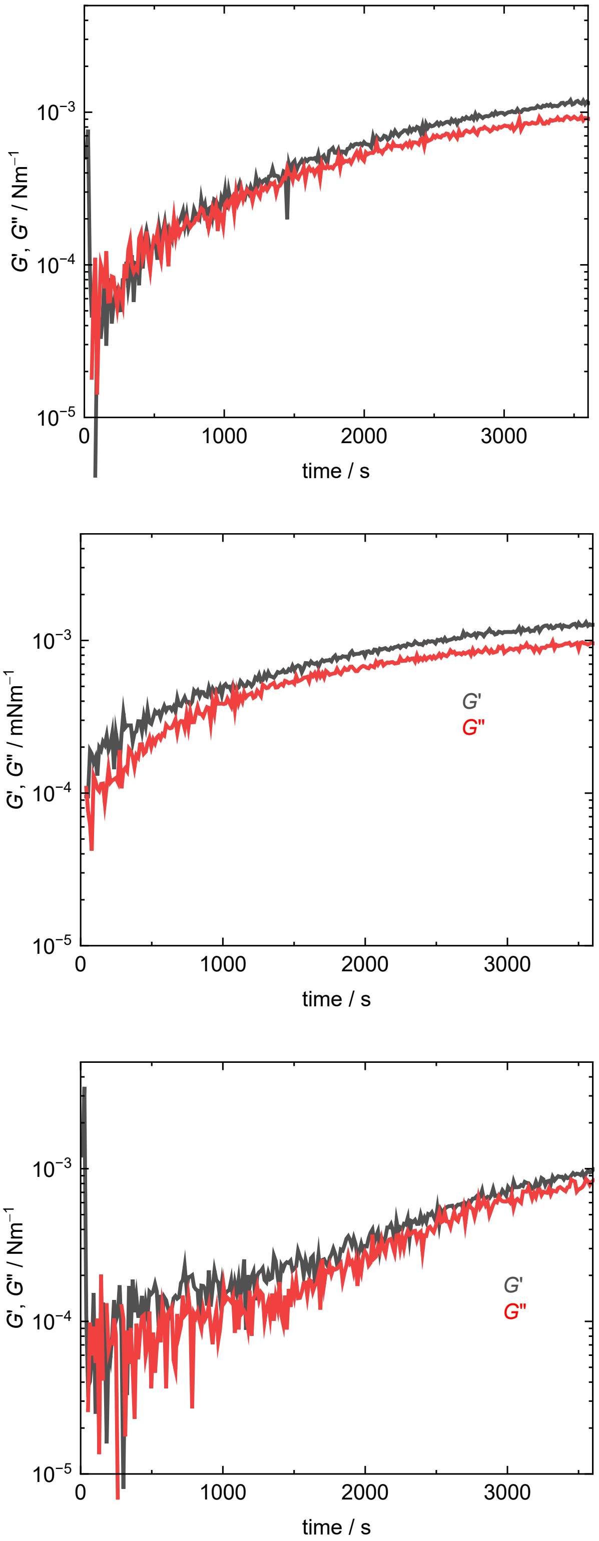

Fig.S5-7 Time evolution of G' (black) and G'' (red) at $E_{\mathrm{F}}^{\mathrm{W}}$ = +0.6 V after the BSA solution was injected. The top one is shown in Fig.4.

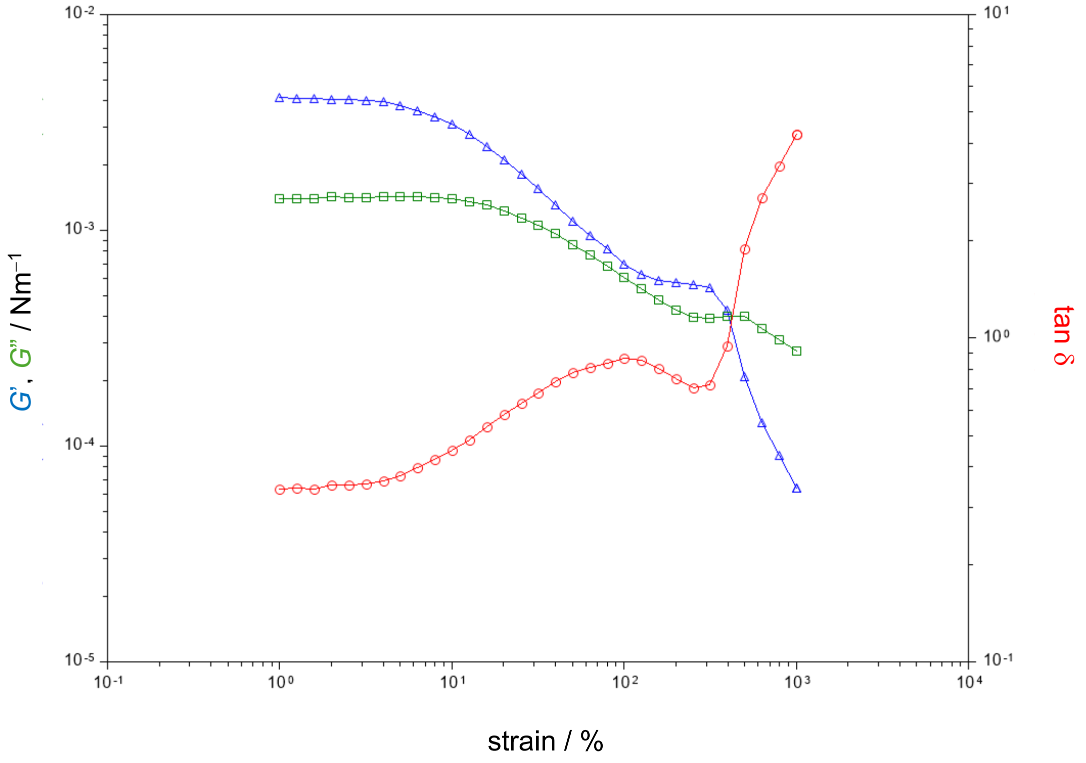

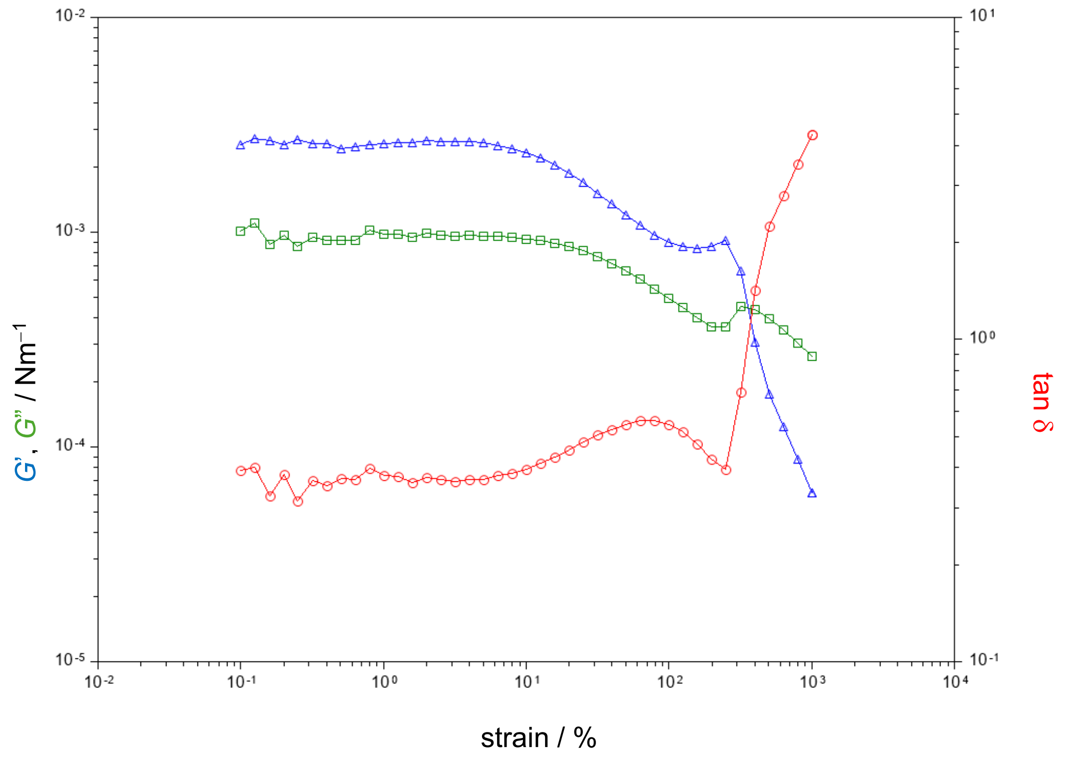

Fig.S5-8a G' (blue line and triangle plots), G'' (green line and square plots) and tanδ (red line and circle plots) of the PNL at E_F^W= –0.6 V as a function of strain 1 h after the BSA solution was injected. G' and G'' were parallel around at strain = 100 %.

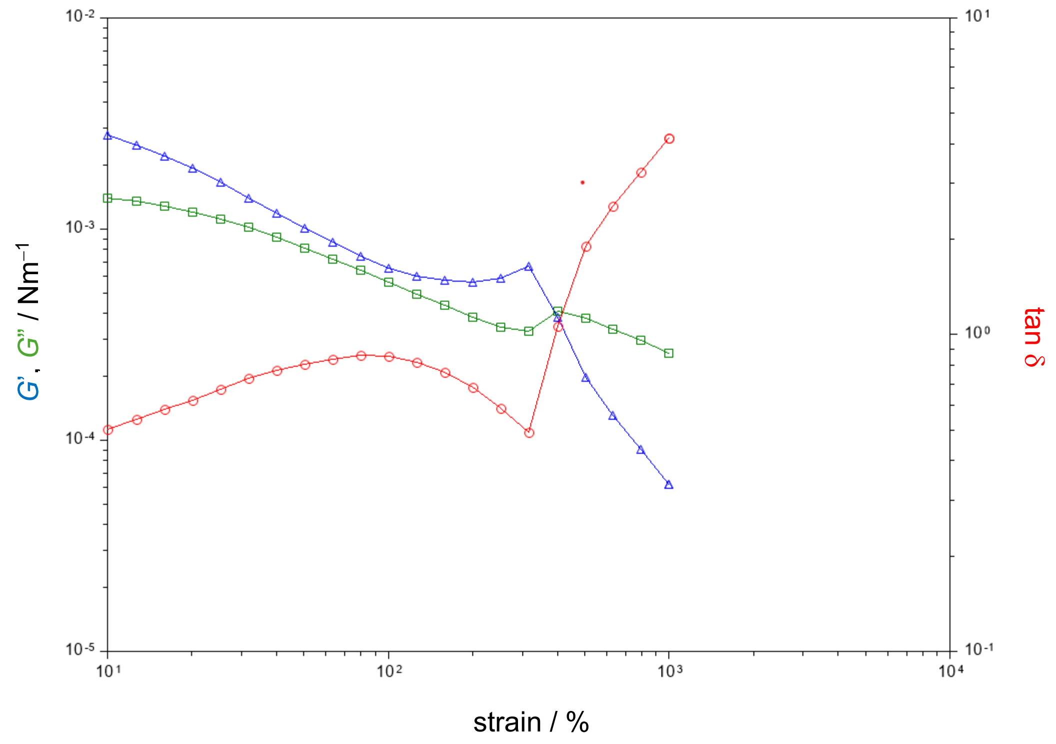

Fig.S5-8b G' (blue line and triangle plots), G'' (green line and square plots) and tanδ (red line and circle plots) of the PNL at E_F^W= –0.6 V as a function of strain 1 h after the BSA solution was injected. G' and G'' were parallel around at strain = 100 %.

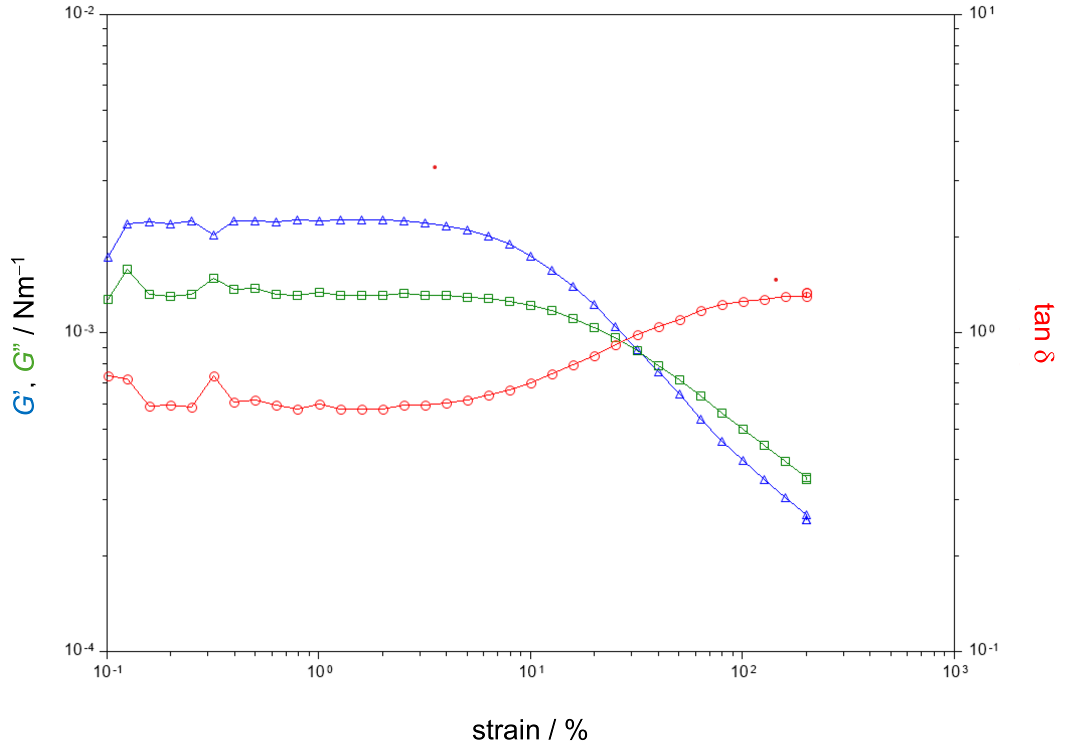

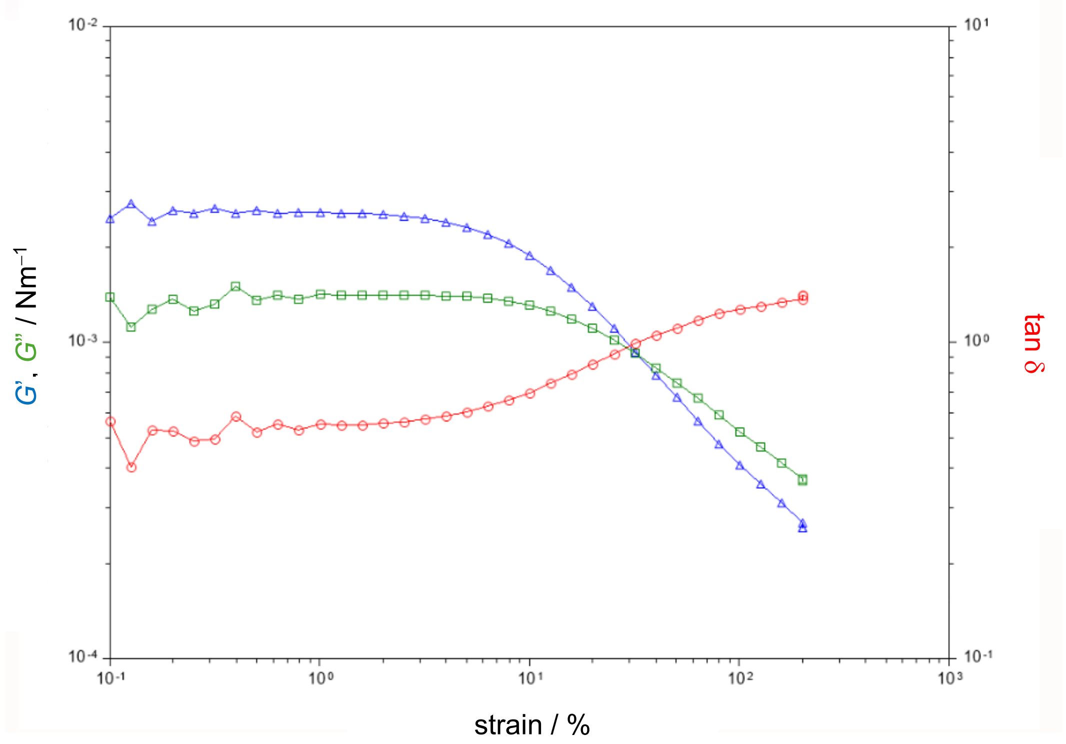

Fig.S5-9a G' (blue line and triangle plots), G'' (green line and square plots) and tanδ (red line and circle plots) of the PNL at $E_{\mathrm{F}}^{\mathrm{W}} = -0.3$ V as a function of strain 1 h after the BSA solution was injected. The top one is shown in Fig.6. The γ_{YP} at $E_{\mathrm{F}}^{\mathrm{W}} = -0.3$ V in Fig.7 was evaluated from the γ_{YP} in Figs.S4-9a and b.

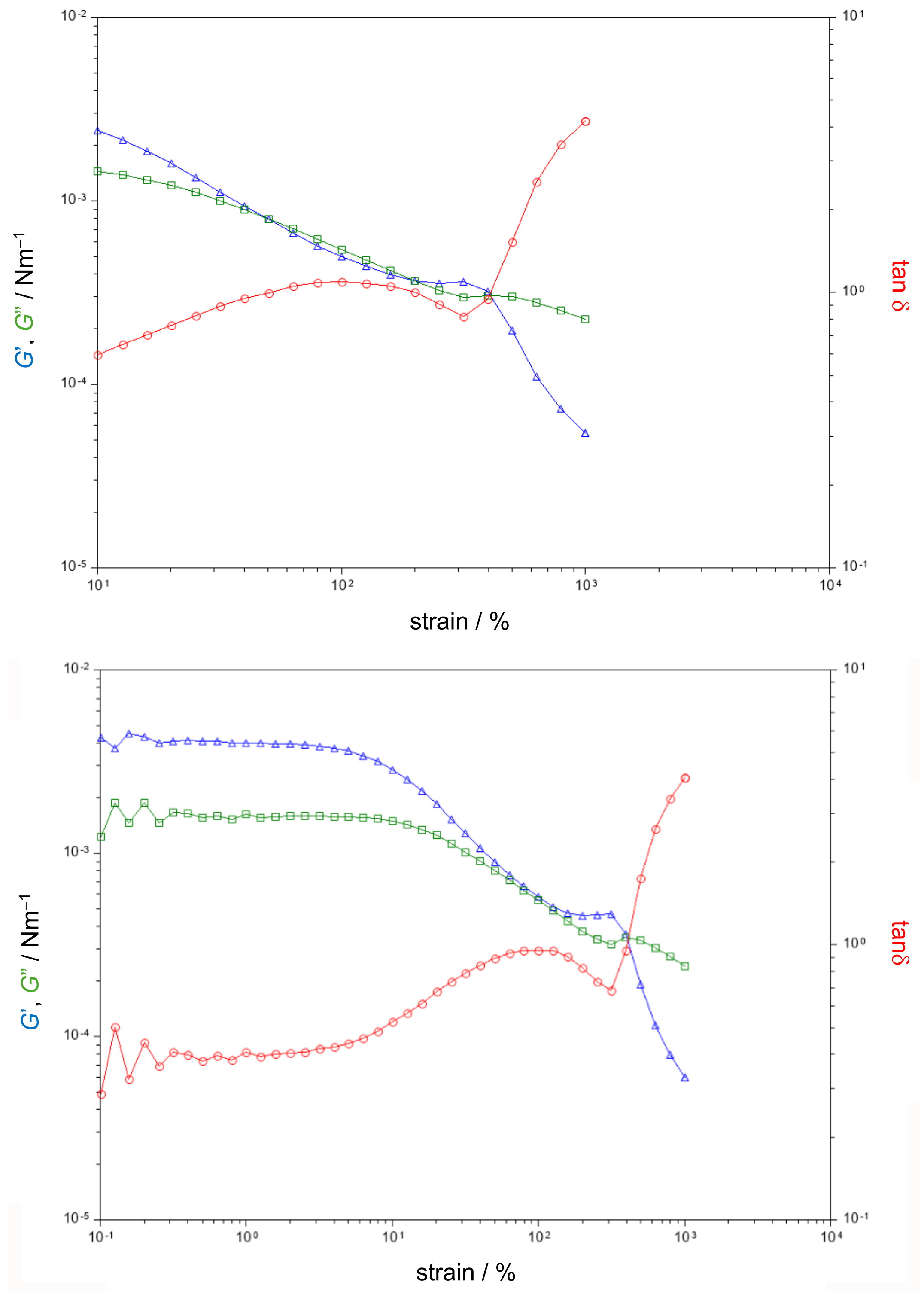

Fig.S5-9b G' (blue line and triangle plots), G'' (green line and square plots) and tanδ (red line and circle plots) of the PNL at $E_{\mathrm{F}}^{\mathrm{W}}$= −0.3 V as a function of strain 1 h after the BSA solution was injected. The γ_{YP} at $E_{\mathrm{F}}^{\mathrm{W}}$ = −0.3 V in Fig.7 was evaluated from the γ_{YP} in Figs.S4-9a and b. In Fig.S4-9b, γ_{YP} in the top graph was determined as the first cross over point at strain γ = 50%, and that in the bottom graph as the cross over point at γ = 340%.

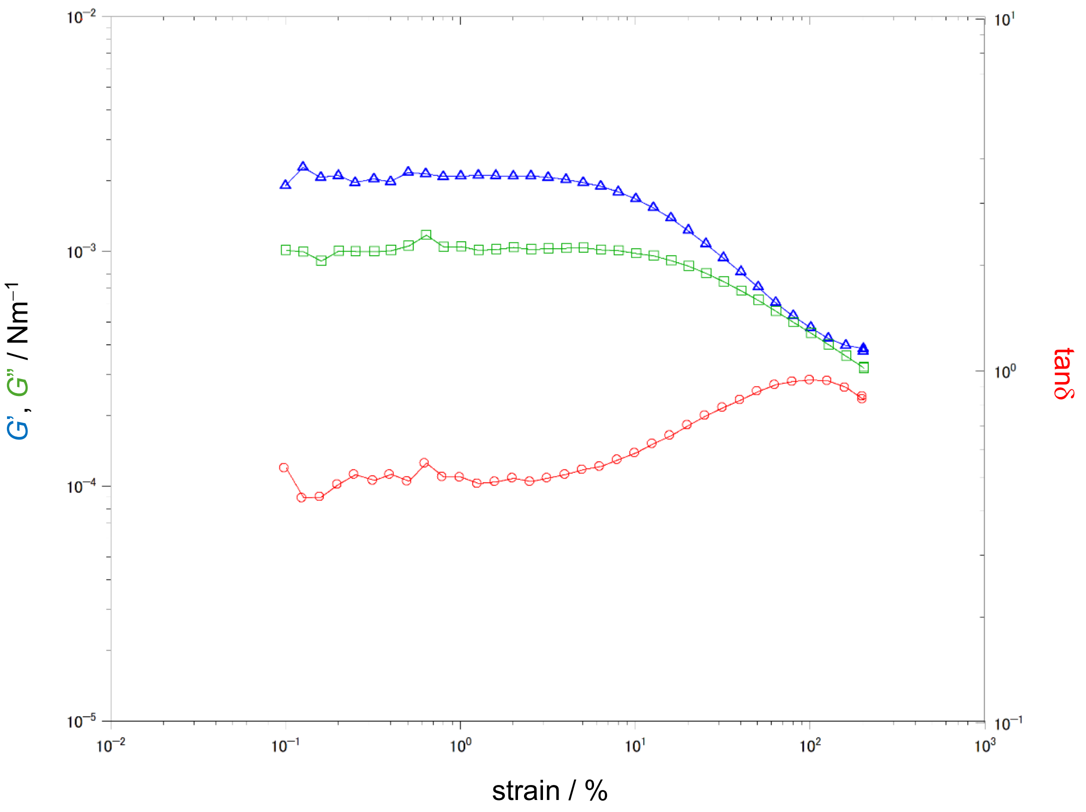

Fig.S5-9c G' (blue line and triangle plots), G'' (green line and square plots) and tanδ (red line and circle plots) of the PNL at E_F^W= –0.3 V as a function of strain 1 h after the BSA solution was injected. G' shows a plateau around 100 % strain. This result was not used to evaluate γ_{YP} at E_F^W = −0.3 V in Fig.7.

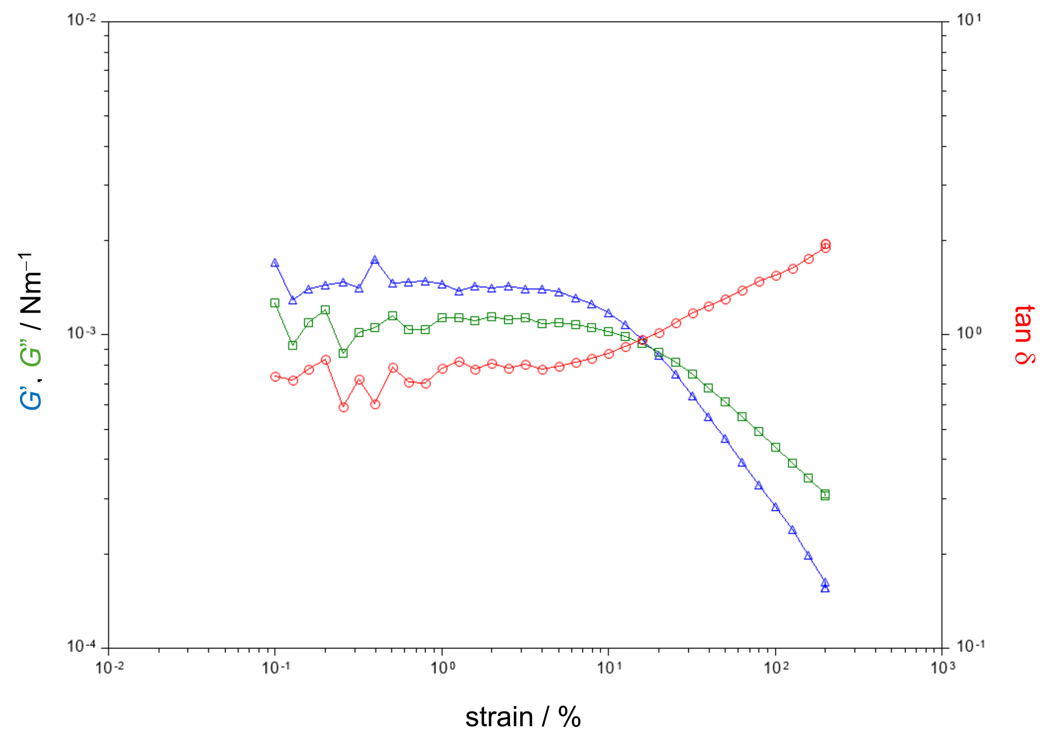

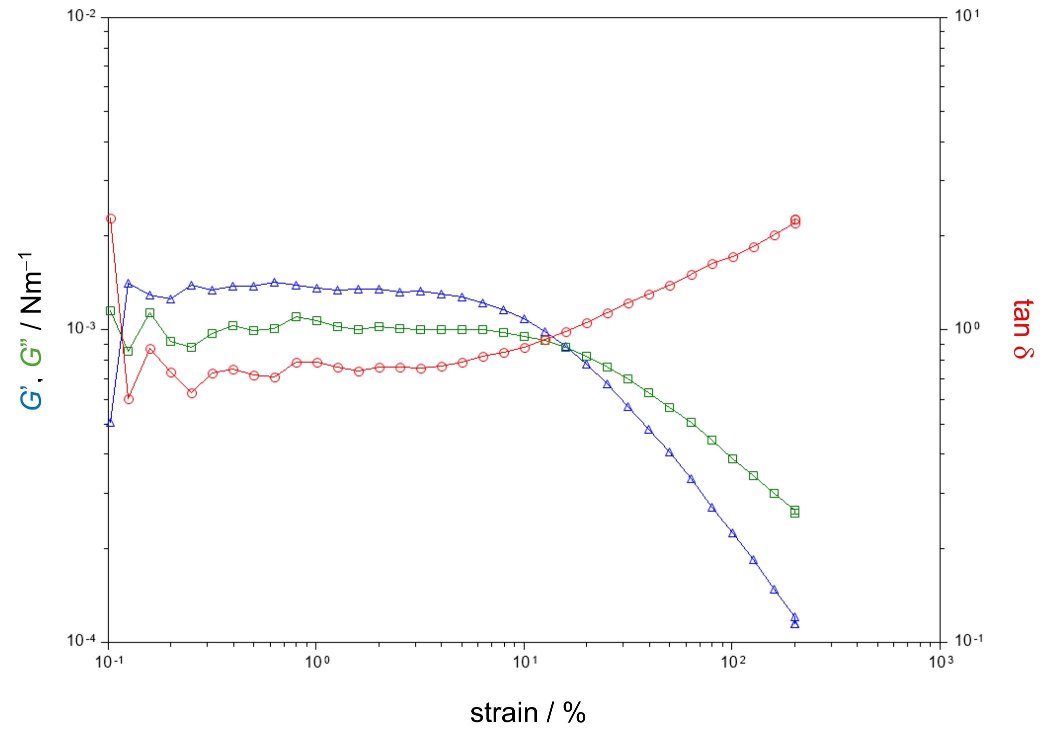

Fig.S5-10a G' (blue line and triangle plots), G'' (green line and square plots) and tanδ (red line and circle plots) of the PNL at $E_{\mathrm{F}}^{\mathrm{W}}$ = 0 V as a function of strain 1 h after the BSA solution was injected.

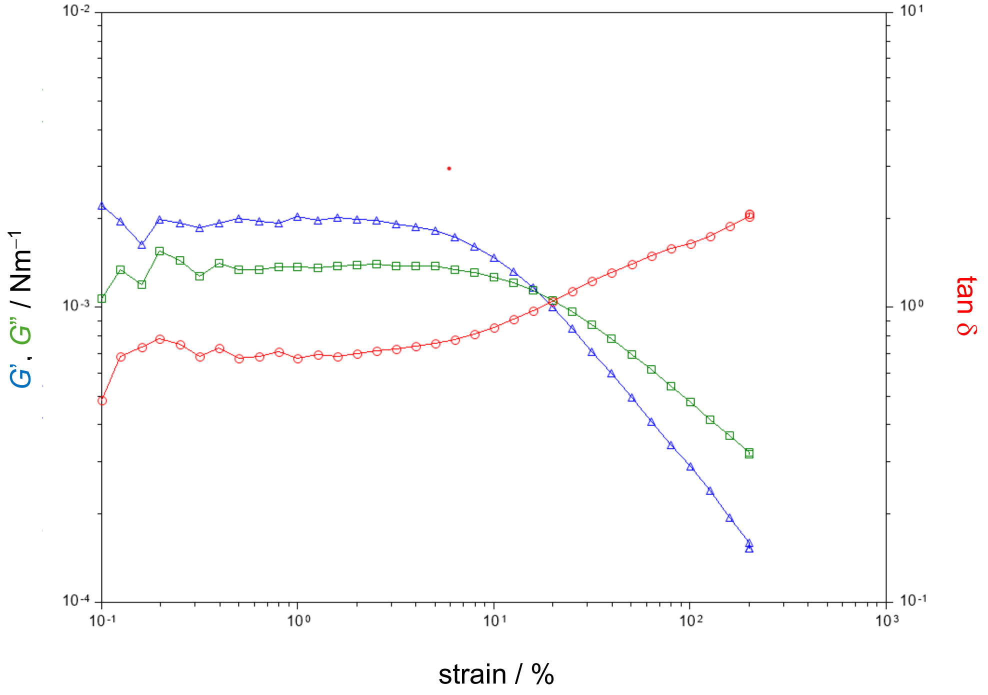

Fig.S5-10b G' (blue line and triangle plots), G'' (green line and square plots) and $\tan\delta$ (red line and circle plots) of the PNL at at E_F^W = 0 V as a function of strain 1 h after the BSA solution was injected. This profile is shown in Fig.6.

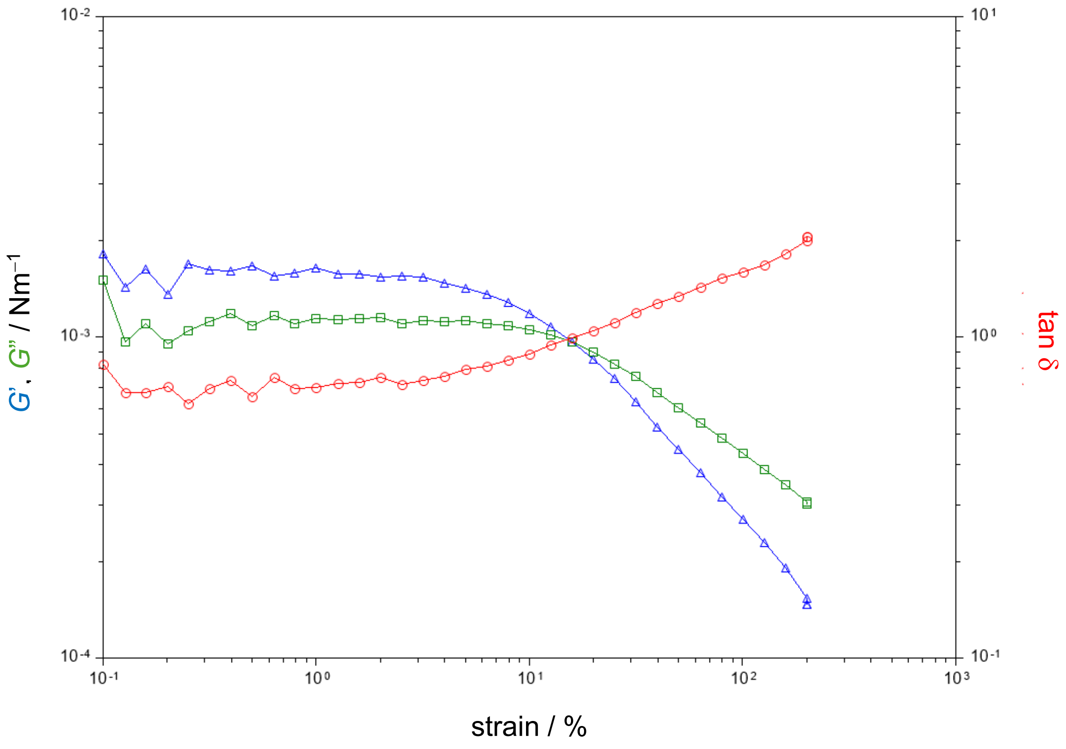

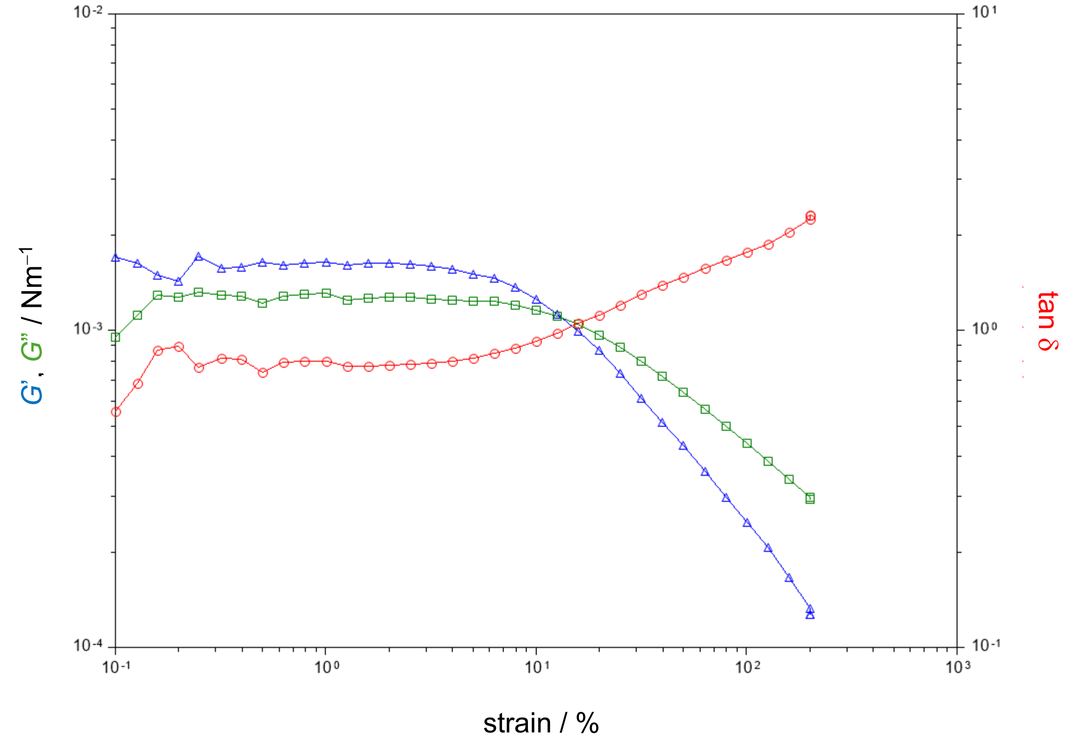

Fig.S5-11a G' (blue line and triangle plots), G'' (green line and square plots) and $\tan\delta$ (red line and circle plots) of the PNL at E_F^W = +0.3 V as a function of strain 1 h after the BSA solution was injected.

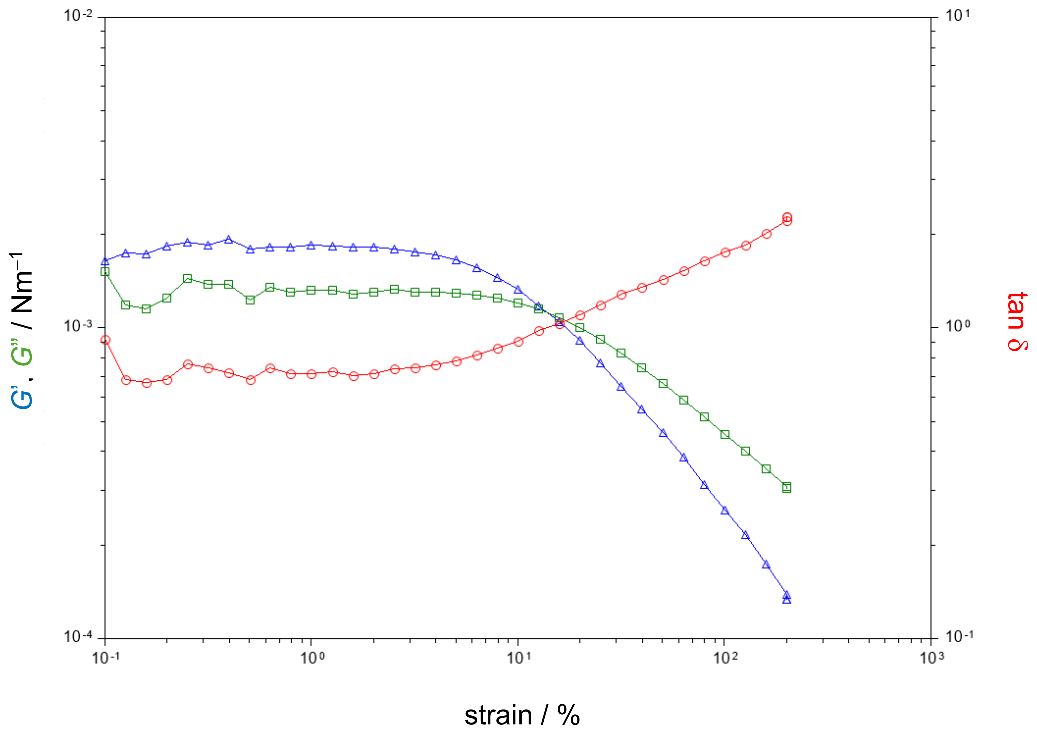

Fig.S5-11b G' (blue line and triangle plots), G'' (green line and square plots) and tanδ (red line and circle plots) of the PNL at E_F^W = +0.3 V as a function of strain 1 h after the BSA solution was injected.

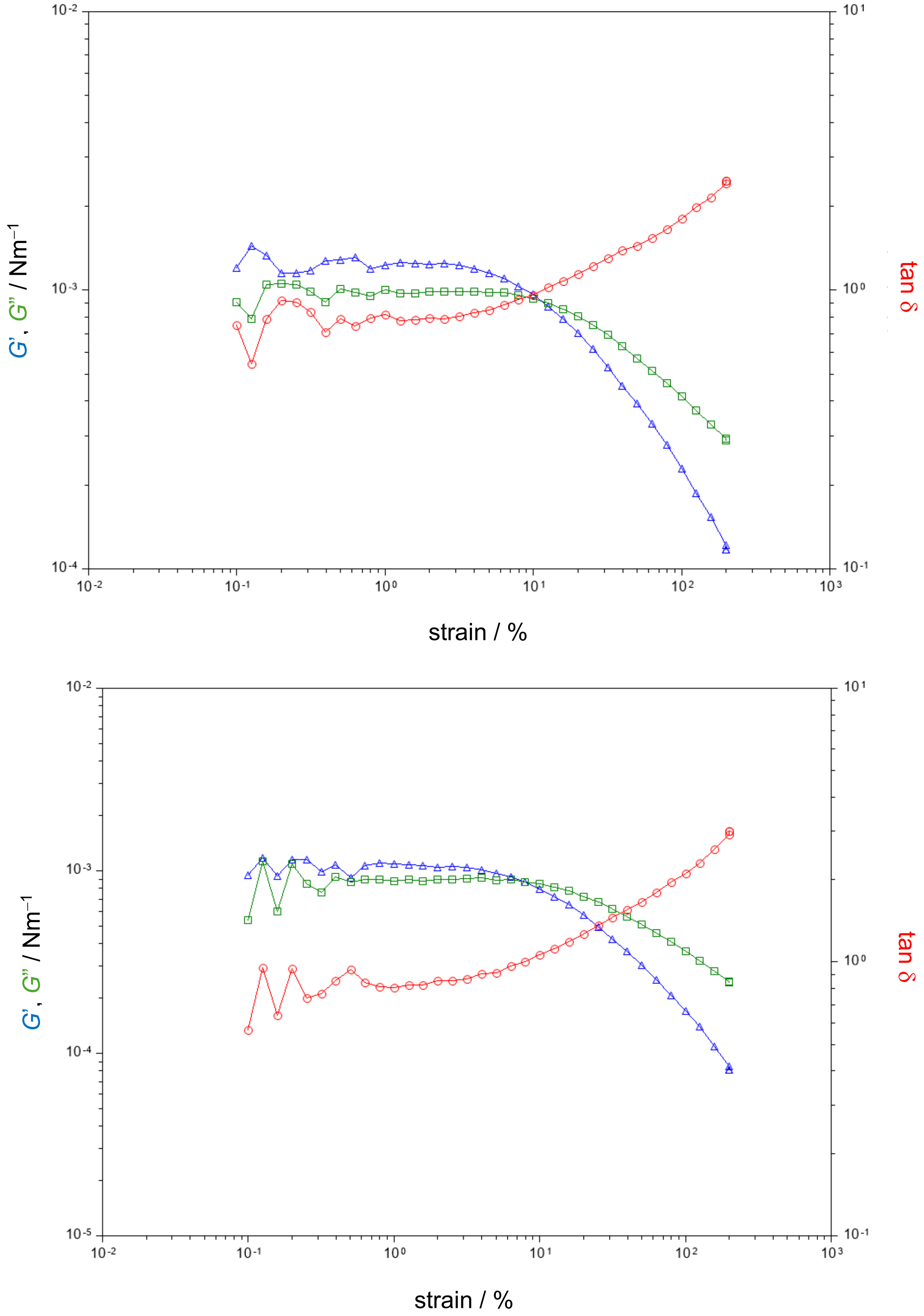

Fig.S5-12a G' (blue line and triangle plots), G'' (green line and square plots) and $\tan\delta$ (red line and circle plots) of the PNL at E_F^W = +0.6 V as a function of strain 1 h after the BSA solution was injected. The top one is shown in Fig.6.

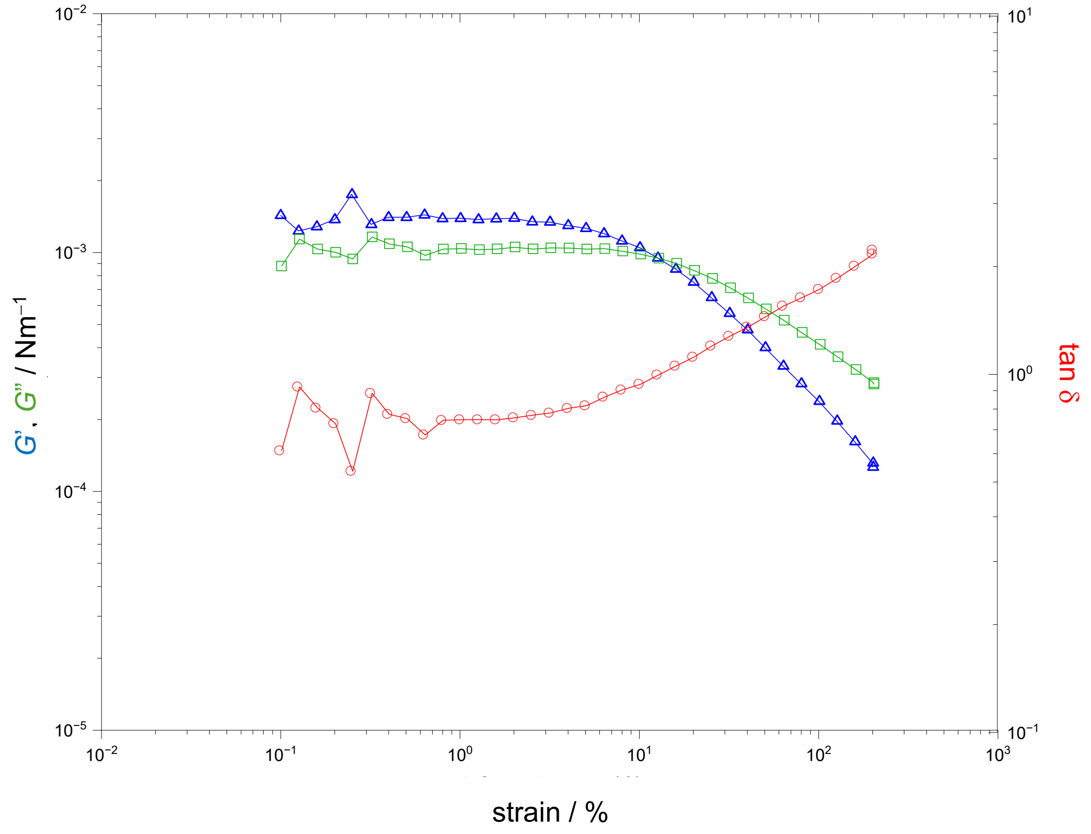

Fig.S5-12b G' (blue line and triangle plots), G'' (green line and square plots) and $\tan\delta$ (red line and circle plots) of the PNL at E_F^W = −0.6 V as a function of strain 1 h after the BSA solution was injected.

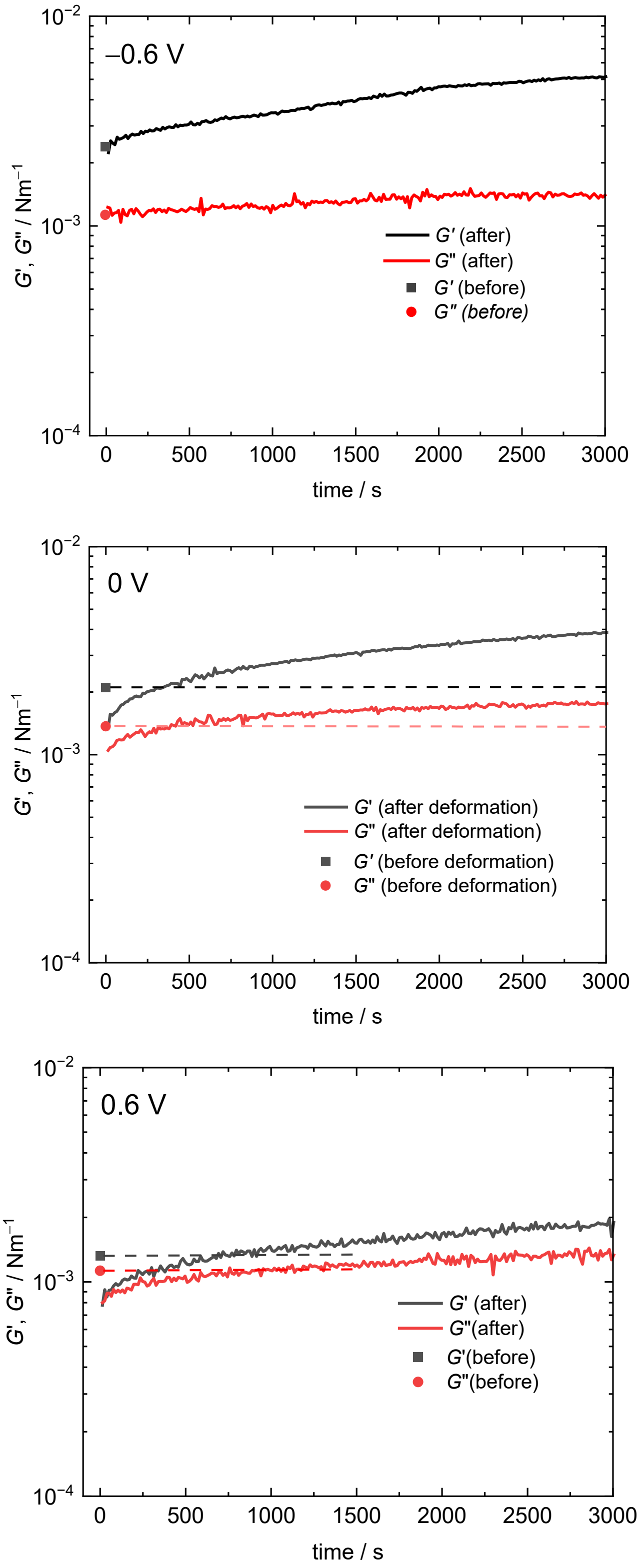

Fig.S5-13 G' and G'' of the cracked PNL at E_F^W = –0.6, 0, and 0.6 V as a function of time at γ = 1 % and ω = 1 Hz. The black square and red circle are the G' and G'' before cracking, respectively. The black and red solid lines are the G' and G'' after cracking, respectively.

References

[1] K. Ishii et al., *Electrochim. Acta*, 513 (2025) 145563.

[2] Y. Kitazumi and T. Kakiuchi, *Langmuir*, 25 (2009) 8062.

[3] S. Katakura et al., *J. Phys. Chem. B*, 124 (2020) 6412.

[4] A. Braslau et al., *Phys. Rev. A*, 38 (1988) 2457.

[5] F.P. Buff et al., *Phys. Rev. Lett.*, 15 (1965) 621.
